# Longitudinal cerebral perfusion in presymptomatic genetic frontotemporal dementia: GENFI results

**DOI:** 10.1002/alz.13750

**Published:** 2024-04-16

**Authors:** Maurice Pasternak, Saira S. Mirza, Nicholas Luciw, Henri J. M. M. Mutsaerts, Jan Petr, David Thomas, David Cash, Martina Bocchetta, Maria Carmela Tartaglia, Sara B. Mitchell, Sandra E. Black, Morris Freedman, David Tang‐Wai, Ekaterina Rogaeva, Lucy L. Russell, Arabella Bouzigues, John C. van Swieten, Lize C. Jiskoot, Harro Seelaar, Robert Laforce, Pietro Tiraboschi, Barbara Borroni, Daniela Galimberti, James B. Rowe, Caroline Graff, Elizabeth Finger, Sandro Sorbi, Alexandre de Mendonça, Chris Butler, Alex Gerhard, Raquel Sanchez‐Valle, Fermin Moreno, Matthis Synofzik, Rik Vandenberghe, Simon Ducharme, Johannes Levin, Markus Otto, Isabel Santana, Antonio P. Strafella, Bradley J. MacIntosh, Jonathan D. Rohrer, Mario Masellis, the GENetic Frontotemporal dementia Initiative (GENFI), Annabel Nelson, Martina Bocchetta, David Cash, David L Thomas, Emily Todd, Hanya Benotmane, Jennifer Nicholas, Kiran Samra, Rachelle Shafei, Carolyn Timberlake, Thomas Cope, Timothy Rittman, Alberto Benussi, Enrico Premi, Roberto Gasparotti, Silvana Archetti, Stefano Gazzina, Andrea Arighi, Chiara Fenoglio, Elio Scarpini, Giorgio Fumagalli, Vittoria Borracci, Giacomina Rossi, Giorgio Giaccone, Giuseppe Di Fede, Paola Caroppo, Pietro Tiraboschi, Sara Prioni, Veronica Redaelli, David Tang‐Wai, Ekaterina Rogaeva, Miguel Castelo‐Branco, Morris Freedman, Ron Keren, Sandra Black, Sara Mitchell, Christen Shoesmith, Robart Bartha, Rosa Rademakers, Jackie Poos, Janne M. Papma, Lucia Giannini, Rick van Minkelen, Yolande Pijnenburg, Benedetta Nacmias, Camilla Ferrari, Cristina Polito, Gemma Lombardi, Valentina Bessi, Michele Veldsman, Christin Andersson, Hakan Thonberg, Linn Öijerstedt, Vesna Jelic, Paul Thompson, Tobias Langheinrich, Albert Lladó, Anna Antonell, Jaume Olives, Mircea Balasa, Nuria Bargalló, Sergi Borrego‐Ecija, Ana Verdelho, Carolina Maruta, Catarina B. Ferreira, Gabriel Miltenberger, Frederico Simões do Couto, Alazne Gabilondo, Ana Gorostidi, Jorge Villanua, Marta Cañada, Mikel Tainta, Miren Zulaica, Myriam Barandiaran, Patricia Alves, Benjamin Bender, Carlo Wilke, Lisa Graf, Annick Vogels, Mathieu Vandenbulcke, Philip Van Damme, Rose Bruffaerts, Koen Poesen, Pedro Rosa‐Neto, Serge Gauthier, Agnès Camuzat, Alexis Brice, Anne Bertrand, Aurélie Funkiewiez, Daisy Rinaldi, Dario Saracino, Olivier Colliot, Sabrina Sayah, Catharina Prix, Elisabeth Wlasich, Olivia Wagemann, Sandra Loosli, Sonja Schönecker, Tobias Hoegen, Jolina Lombardi, Sarah Anderl‐Straub, Adeline Rollin, Gregory Kuchcinski, Maxime Bertoux, Thibaud Lebouvier, Vincent Deramecourt, Beatriz Santiago, Diana Duro, Maria João Leitão, Maria Rosario Almeida, Miguel Tábuas‐Pereira, Sónia Afonso, Valentina Cantoni

**Affiliations:** ^1^ Hurvitz Brain Sciences Program Sunnybrook Research Institute Toronto Ontario Canada; ^2^ Institute of Medical Science Temerty Faculty of Medicine University of Toronto Toronto Ontario Canada; ^3^ Medical Biophysics University of Toronto Toronto Ontario Canada; ^4^ Department of Radiology and Nuclear Medicine Amsterdam Neuroscience Amsterdam University Medical Center Amsterdam the Netherlands; ^5^ Helmholtz‐Zentrum Dresden‐Rossendorf Institute of Radiopharmaceutical Cancer Research Dresden Germany; ^6^ Department of Brain Repair and Rehabilitation UCL Queen Square Institute of Neurology Queen Square London UK; ^7^ Dementia Research Centre Department of Neurodegenerative Disease UCL Queen Square Institute of Neurology Queen Square London UK; ^8^ Tanz Centre for Research in Neurodegenerative Diseases University of Toronto Toronto Ontario Canada; ^9^ Memory Clinic University Health Network Toronto Ontario Canada; ^10^ Division of Neurology Department of Medicine Sunnybrook Health Sciences Centre Toronto Ontario Canada; ^11^ Rotman Research Institute Baycrest Health Sciences Toronto Ontario Canada; ^12^ Cognitive Neurology Research Unit Sunnybrook Health Sciences Centre Toronto Ontario Canada; ^13^ Krembil Research Institute University Health Network Toronto Ontario Canada; ^14^ Department of Neurology Erasmus Medical Center Rotterdam the Netherlands; ^15^ Clinique Interdisciplinaire de Mémoire CHU de Québec, Département des Sciences Neurologiques Université Laval Quebec Quebec Canada; ^16^ Division of Neurology Fondazione IRCCS Istituto Neurologico Carlo Besta Milan Italy; ^17^ Department of Clinical and Experimental Sciences University of Brescia Brescia Italy; ^18^ Department of Biomedical Surgical and Dental Sciences University of Milan Milan Italy; ^19^ Neurodegenerative Diseases Unit Fondazione IRCCS Ca' Granda Ospedale Maggiore Policlinico Milan Italy; ^20^ Department of Clinical Neurosciences University of Cambridge Cambridge UK; ^21^ Cambridge University Hospitals NHS Trust University of Cambridge Cambridge UK; ^22^ Medical Research Council Cognition and Brain Sciences Unit University of Cambridge Cambridge UK; ^23^ Department of Neurobiology Care Sciences and Society Karolinska Institutet Huddinge Sweden; ^24^ Unit for Hereditary Dementias Karolinska University Hospital Solna Sweden; ^25^ Department of Clinical Neurological Sciences Western University London Ontario Canada; ^26^ Department of Neurofarba University of Florence Florence Italy; ^27^ Fondazione Don Carlo Gnocchi Istituto di Ricovero e Cura a Carattere Scientifico Florence Italy; ^28^ Neurology Department Faculty of Medicine University of Lisbon Lisbon Portugal; ^29^ Nuffield Department of Clinical Neurosciences Medical Sciences Division University of Oxford Oxford UK; ^30^ Department of Brain Sciences Imperial College London London UK; ^31^ Division of Psychology Communication and Human Neuroscience Wolfson Molecular Imaging Centre University of Manchester Manchester UK; ^32^ Department of Geriatric Medicine Klinikum Hochsauerland GmbH Arnsberg Germany; ^33^ Department of Nuclear Medicine Center for Translational Neuro‐ and Behavioral Sciences University Hospital Essen Essen Germany; ^34^ Alzheimer's Disease and Other Cognitive Disorders Unit Hospital Clinic de Barcelona Barcelona Spain; ^35^ Cognitive Disorders Unit Department of Neurology Donostia University Hospital Gipuzkoa Building Begiristain Doktorea Pasealekua Donostia–San Sebastian Gipuzkoa Spain; ^36^ Neuroscience Area Biodonostia Health Research Institute Donostia–San Sebastian Gipuzkoa Spain; ^37^ Department of Neurodegenerative Diseases Hertie‐Institute for Clinical Brain Research and Center of Neurology University of Tübingen Tübingen Germany; ^38^ Laboratory for Cognitive Neurology Department of Neurosciences KU Leuven Leuven Belgium; ^39^ Department of Psychiatry Douglas Mental Health University Institute McGill University Montreal Quebec Canada; ^40^ McConnell Brain Imaging Centre Montreal Neurological Institute and Hospital Montreal Quebec Canada; ^41^ Department of Neurology Ludwig‐Maximilians‐University Munich Germany; ^42^ Munich Cluster of Systems Neurology (SyNergy) Munich Germany; ^43^ Department of Neurology University of Ulm Ulm Germany; ^44^ Center for Neuroscience and Cell Biology Faculty of Medicine University of Coimbra Coimbra Portugal; ^45^ Campbell Family Mental Health Research Institute Centre for Addiction and Mental Health Toronto Ontario Canada; ^46^ Edmond J. Safra Parkinson Disease Program & Morton and Gloria Shulman Movement Disorder Unit Toronto Western Hospital UHN Toronto Ontario Canada; ^47^ Department of Medicine Division of Neurology University of Toronto Toronto Ontario Canada; ^48^ Department of Neurodegenerative Disease Dementia Research Centre UCL Queen Square Institute of Neurology London UK; ^49^ Neuroimaging Analysis Centre Department of Brain Repair and Rehabilitation UCL Institute of Neurology, Queen Square London UK; ^50^ UK Dementia Research Institute at University College London UCL Queen Square Institute of Neurology London UK; ^51^ Department of Medical Statistics London School of Hygiene and Tropical Medicine London UK; ^52^ Centre for Neurodegenerative Disorders Department of Clinical and Experimental Sciences University of Brescia Brescia Italy; ^53^ Stroke Unit ASST Brescia Hospital Brescia Italy; ^54^ Neuroradiology Unit University of Brescia Brescia Italy; ^55^ Biotechnology Laboratory Department of Diagnostics ASST Brescia Hospital Brescia Italy; ^56^ Neurology ASST Brescia Hospital Brescia Italy; ^57^ Fondazione IRCCS Ca’ Granda Ospedale Maggiore Policlinico Neurodegenerative Diseases Unit Milan Italy; ^58^ University of Milan Centro Dino Ferrari Milan Italy; ^59^ Fondazione IRCCS Istituto Neurologico Carlo Besta Milano Italy; ^60^ The University Health Network Krembil Research Institute Toronto Canada; ^61^ Tanz Centre for Research in Neurodegenerative Diseases University of Toronto Toronto Canada; ^62^ Faculty of Medicine University of Coimbra Coimbra Portugal; ^63^ Baycrest Health Sciences Rotman Research Institute University of Toronto Toronto Canada; ^64^ The University Health Network Toronto Rehabilitation Institute Toronto Canada; ^65^ Sunnybrook Health Sciences Centre Sunnybrook Research Institute University of Toronto Toronto Canada; ^66^ Department of Clinical Neurological Sciences University of Western Ontario London Ontario Canada; ^67^ Department of Medical Biophysics The University of Western Ontario London Ontario Canada; ^68^ Centre for Functional and Metabolic Mapping Robarts Research Institute The University of Western Ontario London Ontario Canada; ^69^ Center for Molecular Neurology University of Antwerp; ^70^ Department of Clinical Genetics Erasmus Medical Center Rotterdam Netherlands; ^71^ Amsterdam University Medical Centre Amsterdam VUmc Amsterdam Netherlands; ^72^ Department of Neuroscience Psychology, Drug Research and Child Health University of Florence Florence Italy; ^73^ Department of Biomedical Experimental and Clinical Sciences “Mario Serio” Nuclear Medicine Unit University of Florence Florence Italy; ^74^ Department of Clinical Neuroscience Karolinska Institutet Stockholm Sweden; ^75^ Center for Alzheimer Research Division of Neurogeriatrics Karolinska Institutet Stockholm Sweden; ^76^ Center for Alzheimer Research Division of Neurogeriatrics Department of Neurobiology Care Sciences and Society, Bioclinicum Karolinska Institutet Solna Sweden; ^77^ Unit for Hereditary Dementias, Theme Aging Karolinska University Hospital Solna Sweden; ^78^ Division of Clinical Geriatrics Karolinska Institutet Stockholm Sweden; ^79^ Division of Neuroscience and Experimental Psychology Wolfson Molecular Imaging Centre University of Manchester Manchester UK; ^80^ Manchester Centre for Clinical Neurosciences Department of Neurology Salford Royal NHS Foundation Trust Manchester UK; ^81^ Alzheimer's disease and Other Cognitive Disorders Unit Neurology Service Hospital Clínic Barcelona Spain; ^82^ Imaging Diagnostic Center Hospital Clínic Barcelona Spain; ^83^ Department of Neurosciences and Mental Health Centro Hospitalar Lisboa Norte ‐ Hospital de Santa Maria & Faculty of Medicine University of Lisbon Lisbon Portugal; ^84^ Laboratory of Language Research Centro de Estudos Egas Moniz Faculty of Medicine University of Lisbon Lisbon Portugal; ^85^ Laboratory of Neurosciences Faculty of Medicine University of Lisbon Lisbon Portugal; ^86^ Faculty of Medicine University of Lisbon Lisbon Portugal; ^87^ Faculdade de Medicina Universidade Católica Portuguesa; ^88^ Cognitive Disorders Unit Department of Neurology Donostia University Hospital San Sebastian Gipuzkoa Spain; ^89^ Neuroscience Area Biodonostia Health Research Insitute San Sebastian Gipuzkoa Spain; ^90^ OSATEK University of Donostia San Sebastian Gipuzkoa Spain; ^91^ CITA Alzheimer San Sebastian Gipuzkoa Spain; ^92^ Department of Educational Psychology and Psychobiology Faculty of Education International University of La Rioja Logroño Spain; ^93^ Department of Diagnostic and Interventional Neuroradiology University of Tübingen Tübingen Germany; ^94^ Center for Neurodegenerative Diseases (DZNE) Tübingen Germany; ^95^ Department of Human Genetics KU Leuven Leuven Belgium; ^96^ Geriatric Psychiatry Service University Hospitals Leuven Leuven Belgium; ^97^ Neuropsychiatry Department of Neurosciences KU Leuven Leuven Belgium; ^98^ Neurology Service University Hospitals Leuven Leuven Belgium; ^99^ Laboratory for Neurobiology VIB‐KU Leuven Centre for Brain Research Leuven Belgium; ^100^ Department of Biomedical Sciences University of Antwerp Antwerp Belgium; ^101^ Biomedical Research Institute Hasselt University Hasselt Belgium; ^102^ Laboratory for Molecular Neurobiomarker Research KU Leuven Leuven Belgium; ^103^ Translational Neuroimaging Laboratory McGill Centre for Studies in Aging McGill University Montreal Québec Canada; ^104^ Alzheimer Disease Research Unit McGill Centre for Studies in Aging Department of Neurology & Neurosurgery McGill University Montreal Québec Canada; ^105^ Sorbonne Université Paris Brain Institute – Institut du Cerveau – ICM Inserm U1127, CNRS UMR 7225 AP‐HP ‐ Hôpital Pitié‐Salpêtrière Paris France; ^106^ Reference Network for Rare Neurological Diseases (ERN‐RND); ^107^ Centre pour l'Acquisition et le Traitement des Images Institut du Cerveau et la Moelle Paris France; ^108^ Centre de référence des démences rares ou précoces IM2A, Département de Neurologie AP‐HP ‐ Hôpital Pitié‐Salpêtrière Paris France; ^109^ Département de Neurologie AP‐HP ‐ Hôpital Pitié‐Salpêtrière Paris France; ^110^ Neurologische Klinik Ludwig‐Maximilians‐Universität München Munich Germany; ^111^ CHU, CNR‐MAJ, Labex Distalz, LiCEND Lille Lille France; ^112^ Univ Lille Lille France; ^113^ Inserm 1172 Lille France; ^114^ Neurology Department Centro Hospitalar e Universitario de Coimbra Coimbra Portugal; ^115^ Centre of Neurosciences and Cell Biology Universidade de Coimbra Coimbra Portugal; ^116^ Instituto Ciencias Nucleares Aplicadas a Saude Universidade de Coimbra Coimbra Portugal

**Keywords:** arterial spin labeling, cerebral perfusion, frontotemporal dementia, presymptomatic biomarker

## Abstract

**INTRODUCTION:**

Effective longitudinal biomarkers that track disease progression are needed to characterize the presymptomatic phase of genetic frontotemporal dementia (FTD). We investigate the utility of cerebral perfusion as one such biomarker in presymptomatic FTD mutation carriers.

**METHODS:**

We investigated longitudinal profiles of cerebral perfusion using arterial spin labeling magnetic resonance imaging in 42 *C9orf72*, 70 *GRN*, and 31 *MAPT* presymptomatic carriers and 158 non‐carrier controls. Linear mixed effects models assessed perfusion up to 5 years after baseline assessment.

**RESULTS:**

Perfusion decline was evident in all three presymptomatic groups in global gray matter. Each group also featured its own regional pattern of hypoperfusion over time, with the left thalamus common to all groups. Frontal lobe regions featured lower perfusion in those who symptomatically converted versus asymptomatic carriers past their expected age of disease onset.

**DISCUSSION:**

Cerebral perfusion is a potential biomarker for assessing genetic FTD and its genetic subgroups prior to symptom onset.

**Highlights:**

- Gray matter perfusion declines in at‐risk genetic frontotemporal dementia (FTD).
- Regional perfusion decline differs between at‐risk genetic FTD subgroups .
- Hypoperfusion in the left thalamus is common across all presymptomatic groups.
- Converters exhibit greater right frontal hypoperfusion than non‐converters past their expected conversion date.
- Cerebral hypoperfusion is a potential early biomarker of genetic FTD.

## 1 BACKGROUND

Frontotemporal dementia (FTD) comprises a group of clinically and pathologically heterogeneous neurodegenerative disorders featuring regional neuron loss primarily in the frontal and temporal cerebral lobes.^1^ It presents a significant burden on society and is a common cause of young onset dementia with an estimated prevalence being between 15 and 22 cases per 100,000 individuals, approaching that of Alzheimer's disease (AD) in this age group.^2^ There is a strong genetic basis, with up to 20% of all cases stemming from autosomal dominant inheritance in three genes: hexanucleotide repeat expansions in chromosome 9 open reading frame 72 (*C9orf72*), as well as mutations in progranulin (*GRN*) and microtubule‐associated protein tau (*MAPT*),^3^, ^4^ with relative prevalence in that order.^5^

While there are currently no approved disease‐modifying therapies for genetic FTD, several promising drug candidates are being evaluated in clinical trials.^6^, ^7^ Such therapeutics would best be applied at prodromal stages of the disease when irrecoverable neuronal damage has not yet taken place. However, clinical trial design benefits from knowledge of the natural history of disease progression and heterogeneity, which highlights the importance of effective biomarker development to address these challenges. As evidenced in studies focusing on familial AD and Huntington's disease, there are crucial characteristics that effective biomarkers should possess.^8^, ^9^ They should be acquired with relative ease for sustainable longitudinal assessment, reliably change at the presymptomatic stage in a manner that delineates not only individuals at risk of the disease versus those not at risk, but also disease variants. Furthermore, they should identify differences between individuals at risk who eventually develop symptoms versus those who remain asymptomatic, as this would be of particular interest to therapeutics that aim to impede disease progression.

Given the high penetrance of genetic FTD mutations, presymptomatic individuals are a particularly important population for investigating the early signatures of FTD progression and for the identification of disease‐monitoring biomarkers.^3^, ^10^ A variety of studies have provided robust neuroimaging findings on presymptomatic genetic FTD in terms of structural and functional brain changes.^11^, ^12^, ^13^, ^14^, ^15^, ^16^, ^17^, ^18^, ^19^, ^20^, ^21^, ^22^ Assessment of these and other such studies also suggests that functional measures, such as neuronal connectivity, precede structural changes such as atrophy.^5^ There is a need to advance the body of evidence, notably: extending beyond cross‐sectional design to study disease change over time, incorporating all three major mutation variants of genetic FTD, increasing sample size, using non‐invasive imaging techniques that avoid ionizing radiation, and placing a greater focus on presymptomatic carriers as opposed to pooling presymptomatic and symptomatic carriers in comparisons.

We extend upon these previous observations by conducting the largest longitudinal analysis of cerebral perfusion across all three genetic FTD subgroups in presymptomatic individuals at risk for genetic FTD using arterial spin labeling (ASL) magnetic resonance imaging (MRI). ASL is a non‐invasive imaging modality in which an individual's blood is magnetically labeled, thereby acting as an endogenous tracer to measure cerebral perfusion, which in turn is assumed to be tightly coupled to brain metabolism.^23^ This study also highlights the image processing and quality‐control steps necessary for robust cerebral perfusion quantification across multiple study sites and MRI scanners while accounting for partial volume effects stemming from gray matter atrophy.^24^ We have previously demonstrated that regional perfusion delineates presymptomatic FTD carriers from non‐carrier controls in a cross‐sectional study of all three groups in genetic FTD using ASL.^13^

This study investigates regional and global cerebral perfusion changes over time in presymptomatic FTD mutation carriers stratified according to genetic subgroup. We hypothesized that cerebral perfusion would decline over time to a greater extent in one or more of the presymptomatic genetic subgroups relative to non‐carrier controls. Furthermore, the degree of this perfusion decline will differ among brain regions when comparing presymptomatic carrier groups. Finally, perfusion relative to baseline will have declined to a greater extent in presymptomatic carriers who eventually converted into symptomatic FTD versus unaffected carriers who surpassed the time at which they were expected to exhibit symptoms.

## 2 METHODS

### 2.1 Participants

Data were drawn from the fifth data freeze of the Genetic Frontotemporal Dementia Initiative (GENFI) database, with images acquired from 23 sites across Canada, the United Kingdom, Italy, the Netherlands, Sweden, and Portugal between January 30, 2012, and May 31, 2019. Participants were presymptomatic individuals at baseline who were carriers of a genetic mutation in one of the *C9orf72*, *GRN*, or *MAPT* genes, but who had no clinical symptoms of FTD present, as assessed by a trained clinician. Further details regarding the inclusion criteria are listed elsewhere.^20^ Non‐carrier controls were first‐degree relatives of the presymptomatic carriers and who were confirmed to not carry mutations in *C9orf72*, *GRN*, or *MAPT*. Individuals who converted into symptomatic FTD during the study were included in a post hoc, secondary analysis after the primary study was complete (see details below).

### 2.2 Ethics and patient consent

Ethical review boards from all sites approved the study protocol and all participating individuals provided written and informed consent in agreement with the Declaration of Helsinki.

### 2.3 Genotype testing

Verification of *C9orf72*, *GRN*, or *MAPT* mutation being present/absent was done using a standardized protocol at each site. Mutations were detected either by DNA sequencing or allele‐specific polymerase chain reaction (PCR)‐based evaluation of *GRN* or *MAPT*. *C9orf72* hexanucleotide repeat expansions were evaluated using a previously described two‐step genotyping procedure.^25^ Genetic guardians at each site uploaded the mutation results directly to the centralized database. All research personnel and clinicians performing clinical and cognitive/behavioral evaluations, as well as the physical exam, were blinded to mutation status.

### 2.4 MRI image acquisition

T1‐weighted and ASL sequences were collected at the respective sites and image processing steps were taken to enable multi‐site analysis.^20^ Image processing accounted for five main ASL acquisition variants: pseudo‐continuous ASL (PCASL) 2D gradient‐echo echo‐planar imaging (EPI) on 3T Philips Achieva scanners with and without accompanying proton‐density (M0) scans; and pulsed ASL (PASL) 3D gradient‐ and spin‐echo (GRaSE) on 3T Siemens Trio Tim or Prisma Fit machines with or without an accompanying M0 scan, and a PCASL 3D fast‐spin‐echo stack‐of‐spirals on 3T General Electric MR750 scanners with an M0 scan. Detailed ASL parameters are provided in the supporting information (Table S1).

**RESEARCH IN CONTEXT**

1. **Systematic review**: The authors reviewed the literature using PubMed. Cerebral perfusion has shown promise in characterizing genetic frontotemporal dementia (FTD). However, most of the prior literature is cross‐sectional and/or limited to one or two subsets of the major genetic groups (*C9orf72*, *GRN*, and *MAPT*).
2. **Interpretation**: Our study provides evidence that cerebral perfusion may be an early biomarker for assessing at‐risk genetic FTD. Decreases in cerebral perfusion delineate not only all major FTD genetic groups from controls, but also between‐group differences as well. Cerebral perfusion decreases also distinguish converter individuals from older non‐converters.
3. **Future directions**: Measures of cerebral perfusion in early stages of FTD may improve prediction of symptom onset in those genetically at risk. Incorporating cerebral perfusion alongside other imaging measures, such as white matter tract integrity and gray matter atrophy, may significantly improve our understanding of disease mechanisms and can be incorporated into clinical trial design.

### 2.5 ASL image processing

As in our previous cross‐sectional study,^13^ we used ExploreASL software (version 1.10.0),^26^ which is based on the Statistical Parametric Mapping 12 (SPM12) MATLAB package to process ASL scans from the various sites, vendors, and sequences. Briefly, T1‐weighted structural images were segmented into gray and white matter tissue partial volume maps and spatially normalized to a population template in Montreal Neurological Institute 152 standard space using geodesic shooting.^27^ Structural volumes of whole brain gray matter tissues were collected at this time using SPM12 for ancillary structural MRI analysis. Transformation matrices were saved for subsequent application in bringing cerebral perfusion images into a common space for parcellation. ASL time series were corrected for motion outliers using rigid‐body transformation coupled with the Enhancement of Automated Blood Flow Estimates (ENABLE) outlier exclusion algorithm,^28^ followed by pairwise subtraction to produce perfusion‐weighted images (PWI). If M0 images were not acquired at scan time, substitute M0 images were constructed using the mean of the non‐labeled ASL scans without background suppression. M0 volumes were smoothed with a 16 mm full‐width half‐maximum (FWHM) Gaussian kernel to create a bias field that avoided division artifacts during perfusion quantification and canceled out acquisition‐specific B1‐field inhomogeneities. Cerebral perfusion quantification itself followed a single‐compartment model approach and recommendations outlined in the ASL consensus paper.^29^

### 2.6 Quality control and corrections

For cerebral perfusion image quality control, we used the same steps as previously described.^13^ Scans were independently and visually assessed by two authors (M.P., N.L.) with more than 3 years of experience handling ASL data. Scans which featured significant image acquisition or processing issues were discarded such as those with poor signal‐to‐noise, uneven labeling, arterial transit time artifacts, severe motion, distortions from improper coregistration, artifacts, and clipping (see examples in Figure S1 in supporting information). An intraclass correlation score of 0.83 was reached, which is considered “good.”^30^ Remaining inconsistencies were resolved by consensus. In total, 40 participants were excluded during this process. To account for arterial transit time artifacts not immediately evident by visual inspection, images were also assessed quantitatively by their gray matter spatial coefficient of variation,^31^ and excluded if this measure exceeded 0.8. This did not result in any further loss of participant scans.

To adjust for the effects of scans acquired at different sites, as well as differences arising from changes or upgrades to scanner models and software versions between time points within those sites, we performed a group‐based voxel‐wise bias field correction approach. Scans were placed into groupings according to their site, scanner model, and major software version. Groups that contained fewer than four scans were excluded, resulting in the removal of one participant. Scans acquired during visits in which a participant was confirmed by a trained clinician to convert into clinical FTD presentation (*n* = 3) were withheld from contributing to the generation of group‐specific bias fields, as it is advised to estimate bias fields on the basis of individuals without potentially significant pathophysiological alterations.^32^ For each grouping, a mean perfusion image was calculated and smoothed with a 6.4 mm FWHM Gaussian kernel. A grand mean perfusion image for the entire population was then calculated from these individual group means. This grand‐mean image was then rescaled such that the mean gray matter perfusion would be a physiologically reasonable 60 mL/min/100 g value,^33^ which involved rescaling by a factor of 1.14. Bias fields for each grouping were then calculated by dividing its mean perfusion image by the rescaled grand‐mean image. Finally, individual cerebral perfusion images were rescaled by being multiplied against their grouping's bias field image.^13^

To account for the effect of gray matter atrophy, which has been previously demonstrated to be detectable in presymptomatic FTD carriers,^19^ rescaled perfusion images were corrected for partial volume effects (PVE) using a voxel‐wise local linear regression within a 3D Gaussian kernel based on probability tissue maps.^34^ For a secondary analysis (see below), converter scans which were initially withheld from bias field image generation were also rescaled by the appropriate group‐based bias field that they would have otherwise belonged to, followed by PVE correction.

PVE‐corrected images were parcellated using Automated Anatomical Labeling Atlas version 2 (AAL2) within voxels that had a gray matter partial volume ≥ 50%.^35^ Mean perfusion values from parcellated regions were extracted for statistical analysis. Regions which were not covered in all ASL scans, such as the cerebellum, or those with fewer than 100 voxels of positive signal, were excluded from statistical analysis.

### 2.7 Demographic, clinical, and behavioral data analysis

Participants underwent a standardized clinical assessment at each visit. Within this study, we report the Clinical Dementia Rating plus National Alzheimer's Coordinating Center Behavior and Language Domains Rating Scale Frontotemporal Lobar Degeneration (CDR plus NACC FTLD), the FTD Rating Scale score, the revised Cambridge Behavioral Inventory score, and the Mini‐Mental State Examination (MMSE) score. These measures were statistically assessed between non‐carrier controls and the three presymptomatic mutation carrier groups. To account for the effects of cardiovascular risk factors or other neurological/medical diseases on cerebral perfusion, clinical assessment also recorded the absence, recent occurrence, or remote occurrence of seizures, stroke, traumatic brain injury, hypertension, hypercholesterolemia, diabetes mellitus, smoking, excessive alcohol consumption, recreational drug use, and autoimmune disease. These data are presented in Table S2 in supporting information, and statistical comparisons were made across groups at baseline to ensure that they were balanced in terms of these potential perfusion‐altering risk factors/diseases. To determine the impact of participant exclusion during the quality control and bias field correction steps, demographic and clinical variables were also compared between excluded participants and the retained participants.

Categorical variables were assessed either by Pearson chi square test or Fisher exact test depending on whether any given frequency was lower than a count of five. Continuous variables across groups were assessed with a type III analysis of variance and followed up with Tukey post hoc tests if the omnibus *P* value result was below an alpha of 0.05.

### 2.8 Primary linear mixed effects analysis

A total of 158 non‐carrier controls, 42 *C9orf72*, *70 GRN*, and 31 *MAPT* presymptomatic carriers had useable scans for at least two time points (Table S3 in supporting information). For the primary analysis involving regional cerebral perfusion comparison between non‐carrier controls and the three presymptomatic genetic subgroups, we used mixed effects linear models according to the following R‐style formula:

$$\begin{array}{ccc} Perfusion\;∼\; & & Group+Time+Group:Time+Age_{baseline}+Sex\\ & & +CBF_{baseline}+(1\;|\;SubjectID) \end{array}$$

Perfusion is the cerebral perfusion at any given time point in a particular region of interest (ROI) from the AAL2 atlas or of the whole brain gray matter at a probability of at least 50%. Group is a factor variable with the following levels: non‐carrier controls, presymptomatic *C9orf72* carriers, presymptomatic *GRN* carriers, or presymptomatic *MAPT* carriers. Time refers to the exact time from baseline scan measurement, reported in units of years (*β*). The interaction between these two effects is denoted above as Group:Time. Contrasts were encoded such that the control non‐carrier group served as the reference group. Interaction coefficients are therefore reported as comparing a given presymptomatic genetic subgroup versus the control non‐carrier group and are reported as *β_int_* for a given group. To avoid collinearity issues with time from baseline, age at baseline was used as opposed to age at scan date as a covariate. Other covariates included biological sex and the perfusion measured at baseline for the same ROI. A random intercept clustered over participants was included due to the longitudinal nature of the study with repeat image acquisition and familial relatedness of the participants.

The above model was arrived at using a model‐building approach from a more parsimonious model that did not include baseline perfusion as a covariate. Additional models that were initially tested included permitting a random slope across time for each participant, nesting participants within family membership, or a combination of the model alterations. However, these more complex models failed to properly converge regardless of optimization algorithm or otherwise failed to perform better based on Akaike information criterion, Bayesian information criterion, and a log‐likelihood ratio test (Table S4 in supporting information) for model comparisons.

After confirmation of a significant result from the omnibus test mixed effects model, a post hoc analysis was conducted on the model to assess the profile of differences in whole brain gray matter perfusion between each presymptomatic genetic subgroup and non‐carrier controls. This was achieved by estimating the marginal means at baseline assessment and 1‐year intervals after, with contrasts selecting for the effect of perfusion difference between a given presymptomatic group versus non‐carrier controls.

Linear mixed effects analyses were also conducted on structural MRI volumetric data using the following model:

$$\begin{array}{ccc} Volume\;∼\; & & Group+Time+Group:Time+Age_{baseline}+Sex\\ & & +TIV_{baseline}+(1\;|\;SubjectID) \end{array}$$

where volume refers to global gray matter volumes extracted during image processing. *TIV*_baseline_ refers to the total intracranial volume recorded during the baseline scan visit. All other terms and their meanings are akin to the previously described cerebral perfusion model.

Statistical analyses were carried out in R version 4.2.2 (R Foundation for Statistical Computing) and mixed effects models used the *lme4* package for model fitting, the *afex* package for model convergence assessment, and the *emmeans* package for post hoc testing of the whole‐brain gray matter perfusion model.^36^, ^37^, ^38^ Resulting *P* values from multiple testing were adjusted using Bonferroni correction.

### 2.9 Secondary analysis comparing converters to presymptomatic carriers beyond their expected age of disease onset

During participant recruitment, all presymptomatic carriers had a calculated years to expected disease onset (EYO), as covered in a previous GENFI study.^20^ Briefly, the EYO for a given participant was defined as the difference between the age at baseline assessment versus mean age of disease onset within the family for that participant.

To better understand how cerebral perfusion may be related to clinical conversion, we compared mutation carriers who remained asymptomatic past their EYO (*n* = 22) versus mutation carriers who converted into symptomatic FTD during their follow‐up period (*n* = 19). The definition of a converter within this study involved either a clinician's diagnosis and/or otherwise a change in CDR plus NACC FTLD into a score of one or greater. All control non‐carriers or presymptomatic carriers who did not meet these criteria were excluded from this secondary analysis. This secondary analysis did not possess enough statistical power to stratify the converters across the three major genetic subgroups.

To reduce the influence of converter scans having more acquisitions at later time points, which may bias results in favor of demonstrating converter hypoperfusion, analysis was restricted to time point follow‐up three, based on Fisher exact test confirming an equal proportion of visits between the two converter and non‐converter groups (Table S5 in supporting information). This resulted in a removal of three converter scans that took place at follow‐up visit four but with no loss of participants. An analysis of covariance was conducted to ascertain whether the perfusion difference at the final available time point relative to baseline was statistically different between the two groups. The change in perfusion between baseline and the last time point was the dependent variable. Covariates included the participant's age at baseline and their sex. As genetic subgroups had to be pooled together to achieve sufficient statistical power, this secondary analysis was only carried out for ROIs that passed Bonferroni correction in two or more genetic subgroups within the primary analysis. Additionally, as this was a post hoc analysis, correction across multiple tests was not conducted and only uncorrected *P* values are reported.

## 3 RESULTS

### 3.1 Demographic, clinical, and behavioral data

For demographic variables (Table 1), there were no significant differences between healthy controls and the three genetic FTD subgroups across education, proportions of sex, or proportions of handedness. However, age was statistically different (*F*[3, 297] = 9.98; *P* < 0.001) among the groups and post hoc analysis for age demonstrated that presymptomatic *MAPT* mutation carriers were statistically younger than the non‐carrier control population as well as the *GRN* presymptomatic carrier group. Clinical and behavioral measures (Table 1), including the CDR plus NACC FTLD, the FTD Rating Scale, the Cambridge Behavioral Inventory, and the MMSE were similar between presymptomatic genetic subgroups and non‐carrier controls. Likewise, frequency analysis of cardiovascular risk factors and other neurological/medical diseases that could potentially influence cerebral perfusion (Table S2) found no differences between any participant groups at baseline.

**TABLE 1:** Demographic, structural imaging, and clinical characteristics (*n* = 301).

	Group				
Characteristic	Non‐carrier controls, *n* = 158	*C9orf72* presymptomatic carriers, *n* = 42	*GRN* presymptomatic carriers, *n* = 70	*MAPT* presymptomatic carriers, *n* = 31	*P* value
**Demographics**					
**Age (years)**	46.6 ± 13.0	41.7 ± 10.0	47.4 ± 11.9	38.4 ± 8.9	**<0.001**
**Education (years)**	14.7 ± 3.5	15.1 ± 2.5	15.2 ± 3.5	14.8 ± 3.1	0.70
**Sex**					0.71
Female	99 (63%)	30 (71%)	43 (61%)	19 (61%)	
Male	59 (37%)	12 (29%)	27 (39%)	12 (39%)	
**Handedness**					0.10
Left	8 (5.1%)	2 (4.8%)	10 (14%)	4 (13%)	
Right	149 (94%)	39 (93%)	59 (84%)	27 (87%)	
Other	1 (0.6%)	1 (2.4%)	1 (1.4%)	0 (0%)	
**Clinical measures**					
**CDR plus NACC FTLD score (categories** ^*^)	0 (IQR 0–0)	0 (IQR 0–0)	0 (IQR 0–0)	0 (IQR 0–0)	–
**FTD rating scale (/100)**	96.4 ± 5.9	95.0 ± 8.0	95.5 ± 10.8	93.5 ± 10.3	0.37
**Cambridge Behavioral Inventory (/180)**	4.7 ± 6.1	6.4 ± 7.9	3.8 ± 6.7	7.4 ± 11.0	0.08
**Mini‐Mental State Examination (/30)**	29.4 ± 1.0	29.7 ± 0.6	29.5 ± 1.0	29.7 ± 1.1	0.26
**Structural imaging**					
**Gray matter (mm^3^)**	638 ± 2.4	618 ± 4.6	636 ± 3.6	638 ± 5.4	**<0.001**

*Notes*: For demographic and clinical measures, data are represented as either *n* (%), mean ± standard deviation, or median. *P* values stem from type III analysis of variance for continuous variables and χ^2^ or Fisher exact tests for categorical variables, depending on whether all cells were > 5 or not, respectively. For structural measures, data are represented as group estimate ± standard error based on estimated marginal means extracted from the longitudinal linear mixed effects models. Bold emphasis has been placed on *P* values that are ≤ 0.05.

Abbreviations: *C9orf72*, chromosome 9 open reading frame 72; CDR, Clinical Dementia Rating; FTD, frontotemporal dementia; *GRN*, progranulin; IQR, interquartile range; *MAPT*, microtubule‐associated protein tau; NACC FTLD, National Alzheimer's Coordinating Center Behavior and Language Domains Rating Scale.

* Clinical Dementia Rating plus National Alzheimer's Coordinating Center Behavior and Language Domains Rating Scale Frontotemporal Lobar Degneration categories: 0 (normal); 0.5 (very mild); 1 (mild); 2 (moderate); 3 (severe).

The comparison between excluded participants and retained participants (Table S6 in supporting information) demonstrated that the two populations were statistically comparable for demographic and clinical measures, apart from sex frequencies being significantly different (*χ^2^* [1] = 8.02; *P* < 0.001), with an increased proportion of male individuals featured in the excluded group.

### 3.2 Global gray matter perfusion changes

**FIGURE 1:**
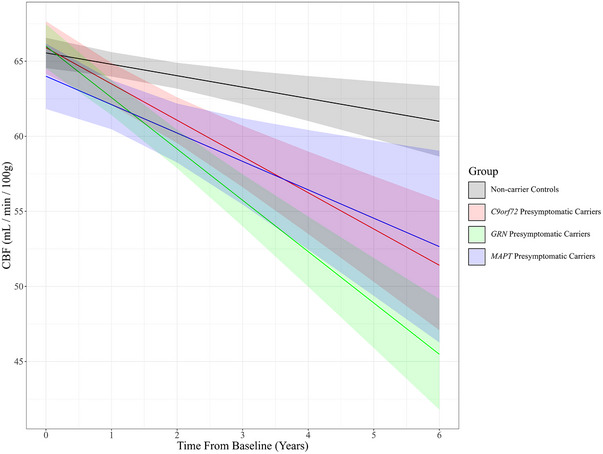
Mixed effects interaction plot of whole brain gray matter perfusion as a function of time from baseline assessment for non‐carrier controls (black) versus presymptomatic carriers of mutations *C9orf72* (orange)*, GRN* (green), and *MAPT* (cyan). Shaded areas represent 95% confidence intervals. *C9orf72*, chromosome 9 open reading frame 72; CBF, cerebral blood flow; *GRN*, progranulin; *MAPT*, microtubule‐associated protein tau.

Global gray matter longitudinal perfusion profiles (Figure 1) demonstrated an overall decreasing trend in all participant groups as a function of time from baseline (non‐carriers = −0.76 ± 0.24 mL/min/100 g/year, *P* = 0.002; *C9orf72* = −2.42 mL/min/100 g/year, *P* < 0.001; *GRN* = −3.42 ± 0.38 mL/min/100 g/year, *P* < 0.001; *MAPT* = −1.89 mL/min/100 g/year, *P* = 0.003), in agreement with the general observation that perfusion decreases with age.^39^ All three presymptomatic genetic subgroups demonstrated a more pronounced rate of perfusion decline relative to non‐carrier controls, albeit to varying degrees. The greatest rate of perfusion decline relative to non‐carrier controls was demonstrated by the *GRN* group (*β_int_* = −2.4 ± 0.5 mL/min/100 g/year; *t*[555] = −4.9; *P* < 0.001), followed by *C9orf72* (*β_int_* = −1.5 ± 0.6 mL/min/100 g/year; *t*[579] = −2.6; *P* = 0.009) and finally the *MAPT* group (*β_int_* = −1.1 ± 0.7 mL/min/100 g/year; *t*[542] = −1.4; *P *= 0.15).

Post hoc analysis of the global perfusion model (Table 2) illustrated that lowered perfusion relative to non‐carrier controls could be identified as early as 1 year after baseline assessment in both *GRN* (−2.22 ± 0.65 mL/min/100 g; *P* = 0.002) and *MAPT* (−2.69 ± 0.90 mL/min/100 g; *P* = 0.009) groups. Both groups also maintained significant lowered perfusion as late as 6 years post‐baseline assessment. The *C9orf72* group also demonstrated significantly lowered global gray matter perfusion relative to controls by 2 years after baseline assessment (−2.96 ± 0.86 mL/min/100 g; *P* = 0.002) and maintained this significant difference onward.

**TABLE 2:** Post hoc marginal means estimation of whole brain gray matter perfusion differences between presymptomatic carriers versus non‐carrier controls at fixed time points.

Time from baseline (years)	*C9orf72* presymptomatic carriers minus non‐carrier controls			*GRN* presymptomatic carriers minus non‐carrier controls			*MAPT* presymptomatic carriers minus non‐carrier controls		
	Estimate (mL/min/100 g)	*df*/*T*‐ratio	*P* value	Estimate (mL/min/100 g)	*df*/*T*‐ratio	*P* value	Estimate (mL/min/100 g)	*df*/*T*‐ratio	*P* value
**0**	0.35 ± 1.01	631.2/0.35	1	0.44 ± 0.85	653.03/0.52	1	−1.55 ± 1.19	673.47/−1.3	0.580
**1**	−1.31 ± 0.79	301.59/−1.66	0.291	−2.22 ± 0.65	306.74/−3.42	**0.002**	−2.69 ± 0.90	303.33/−2.99	**0.009**
**2**	−2.96 ± 0.86	273.04/−3.46	**0.002**	−4.88 ± 0.72	337.04/−6.75	**<0.001**	−3.82 ± 1.07	432.75/−3.56	**0.001**
**3**	−4.62 ± 1.16	452.39/−3.97	**<0.001**	−7.54 ± 1.01	607.45/−7.45	**<0.001**	−4.95 ± 1.56	741.42/−3.17	**0.005**
**4**	−6.28 ± 1.58	627.34/−3.98	**<0.001**	−10.2 ± 1.39	762.33/−7.35	**<0.001**	−6.08 ± 2.17	798.69/−2.81	**0.015**
**5**	−7.93 ± 2.03	720.62/−3.91	**<0.001**	−12.86 ± 1.8	797.19/−7.16	**<0.001**	−7.21 ± 2.81	779.11/−2.57	**0.031**

*Note*: Data derived from the primary analysis linear mixed effects model for whole brain gray matter perfusion. Marginal mean estimates represented as mean ± standard error. *P* values were adjusted using Bonferroni correction. Bold emphasis has been placed on *P* values that are ≤ 0.05 after Bonferroni correction.

Abbreviations: *C9orf72*, chromosome 9 open reading frame 72; *df*, degree of freedom; *GRN*, progranulin; *MAPT*, microtubule‐associated protein tau.

### 3.3 Global gray matter structural changes

Measures extracted from linear mixed effects models of global tissue volumes (Table 1) demonstrated that only the *C9orf72* group featured a measurable degree of gray matter atrophy as early as baseline relative to non‐carrier controls (−19.3 ± 5.12 mm^3^; *P *= 0.002), with no significant differences in gray matter volume found for *GRN* (−1.1 ± 4.2 mm^3^; *P* = 0.8) or *MAPT* (0.7 ± 5.9 mm^3^; *P* = 0.9). However, no significant interaction was observed between the group effect and time (Figure S2 in supporting information), indicating no significant differences in the rates of global gray matter change within the approximate 5‐year period for this population.

### 3.4 Regional perfusion changes

**FIGURE 2:**
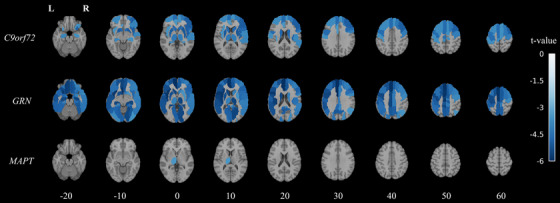
Statistical axial slice maps derived from the mixed effects region of interest analysis examining the interaction effect between mutation status (presymptomatic carrier versus non‐carrier control) and time from baseline for each of the carrier groups. Colors represent Satterthwaite‐approximated *t* values from regions which survived familywise error correction at *P* value < 0.05. Images are shown in neurological display convention, overlaid on top of the Montreal Neurological Institute 152 T1‐weighted template image. *C9orf72*, chromosome 9 open reading frame 72; *GRN*, progranulin; L, left; *MAPT*, microtubule‐associated protein tau; R, right.

Tables 3 and 4 show the coefficients for the main effect of time from baseline (*β*) as well as the interaction coefficients (*β_int_*) for each of the three presymptomatic genetic subgroups relative to the reference non‐carrier control group. Negative values indicate a more pronounced decline in perfusion over time relative to non‐carrier controls. Figure 2 represents the regions that survived Bonferroni correction as *t* values overlaid upon axial brain slices.

**TABLE 3:** Longitudinal region of interest analysis comparing cerebral perfusion between presymptomatic carriers of each major FTD mutation and non‐carrier controls modeled based on time from baseline and its interaction with carrier group within the left hemisphere.

Region			*C9orf72*		*GRN*		*MAPT*	
	*β*	*P*	*β_int_*	*P_int_*	*β_int_*	*P_int_*	*β_int_*	*P_int_*
**Amygdala**	−0.37 ± 0.3	1	−1.38 ± 0.62	1	−2.66 ± 0.55	**<0.001**	−0.86 ± 0.86	1
**Angular gyrus**	−0.82 ± 0.37	1	−1.62 ± 0.78	1	−3.26 ± 0.69	**<0.001**	−0.25 ± 1.07	1
**Anterior cingulate cortex**	−0.57 ± 0.35	1	−2.55 ± 0.73	**0.045**	−3.97 ± 0.65	**<0.001**	−0.8 ± 1.01	1
**Anterior orbitofrontal cortex**	−1.69 ± 0.34	**<0.001**	−1.1 ± 0.7	1	−2.77 ± 0.63	**0.001**	0.23 ± 0.97	1
**Calcarine fissure**	−1.08 ± 0.43	1	−1.18 ± 0.89	1	−3.87 ± 0.79	**<0.001**	−2.62 ± 1.22	1
**Caudate nucleus**	−0.45 ± 0.27	1	−2.61 ± 0.56	**<0.001**	−2.7 ± 0.49	**<0.001**	−0.79 ± 0.77	1
**Dorsolateral superior frontal gyrus**	−1.28 ± 0.33	**0.012**	−2.62 ± 0.7	**0.016**	−3.27 ± 0.62	**<0.001**	−0.71 ± 0.96	1
**Fusiform gyrus**	−0.54 ± 0.3	1	−1.0 ± 0.62	1	−2.68 ± 0.56	**<0.001**	−1.39 ± 0.87	1
**Gyrus rectus**	−0.17 ± 0.35	1	−1.52 ± 0.74	1	−3.72 ± 0.66	**<0.001**	−0.64 ± 1.03	1
**Heschl's gyrus**	−0.33 ± 0.38	1	−2.65 ± 0.79	0.072	−3.96 ± 0.7	**<0.001**	−1.32 ± 1.08	1
**Hippocampus**	−0.39 ± 0.28	1	−2.32 ± 0.59	**0.009**	−2.88 ± 0.53	**<0.001**	−1.55 ± 0.83	1
**Inferior frontal gyrus pars opercularis**	−0.56 ± 0.36	1	−2.54 ± 0.74	0.061	−3.81 ± 0.66	**<0.001**	−1.51 ± 1.02	1
**Inferior frontal gyrus pars orbitalis**	−1.11 ± 0.36	0.175	−1.45 ± 0.75	1	−3.01 ± 0.66	**<0.001**	−1.28 ± 1.03	1
**Inferior frontal gyrus pars triangularis**	−0.69 ± 0.35	1	−2.07 ± 0.74	0.454	−3.47 ± 0.65	**<0.001**	−1.31 ± 1.01	1
**Inferior occipital gyrus**	−0.95 ± 0.4	1	−0.68 ± 0.84	1	−2.84 ± 0.75	**0.014**	−0.59 ± 1.15	1
**Inferior parietal gyrus**	−1.06 ± 0.36	0.261	−1.34 ± 0.74	1	−3.01 ± 0.66	**<0.001**	−0.44 ± 1.02	1
**Inferior temporal gyrus**	−0.83 ± 0.27	0.167	0.18 ± 0.56	1	−2.41 ± 0.49	**<0.001**	−0.62 ± 0.76	1
**Insular cortex**	−0.46 ± 0.31	1	−2.34 ± 0.66	**0.035**	−3.4 ± 0.58	**<0.001**	−0.97 ± 0.9	1
**Lateral orbitofrontal cortex**	−1.4 ± 0.39	**0.036**	−0.67 ± 0.81	1	−2.52 ± 0.72	**0.045**	−0.81 ± 1.12	1
**Lingual gyrus**	−0.48 ± 0.36	1	−2.0 ± 0.75	0.729	−3.31 ± 0.67	**<0.001**	−2.37 ± 1.04	1
**Medial orbitofrontal cortex**	−0.66 ± 0.3	1	−1.01 ± 0.64	1	−3.24 ± 0.57	**<0.001**	−0.26 ± 0.88	1
**Medial superior frontal gyrus**	−0.63 ± 0.31	1	−3.03 ± 0.65	**<0.001**	−3.53 ± 0.58	**<0.001**	−0.9 ± 0.9	1
**Medial‐orbital superior frontal gyrus**	−0.83 ± 0.36	1	−1.88 ± 0.76	1	−3.77 ± 0.68	**<0.001**	0.27 ± 1.05	1
**Middle cingulate cortex**	−0.97 ± 0.32	0.214	−1.94 ± 0.67	0.335	−3.26 ± 0.59	**<0.001**	−0.73 ± 0.91	1
**Middle frontal gyrus**	−1.23 ± 0.35	**0.047**	−2.79 ± 0.74	**0.014**	−2.96 ± 0.65	**<0.001**	−0.98 ± 1.02	1
**Middle occipital gyrus**	−1.3 ± 0.4	0.096	−0.76 ± 0.83	1	−2.41 ± 0.74	0.099	−0.23 ± 1.14	1
**Middle temporal gyrus**	−0.7 ± 0.3	1	−1.09 ± 0.62	1	−2.87 ± 0.55	**<0.001**	0.16 ± 0.86	1
**Middle temporal pole**	−0.52 ± 0.34	1	1.37 ± 0.7	1	−2.06 ± 0.63	0.095	−1.52 ± 0.97	1
**Olfactory cortex**	0.24 ± 0.35	1	−2.12 ± 0.74	0.369	−3.97 ± 0.66	**<0.001**	−0.73 ± 1.03	1
**Paracentral lobule**	−0.92 ± 0.33	0.478	−2.7 ± 0.69	**0.009**	−3.1 ± 0.61	**<0.001**	−0.51 ± 0.94	1
**Parahippocampal gyrus**	−0.37 ± 0.29	1	−0.75 ± 0.61	1	−2.28 ± 0.54	**0.003**	−1.04 ± 0.85	1
**Postcentral gyrus**	−0.71 ± 0.32	1	−2.47 ± 0.67	**0.022**	−2.92 ± 0.59	**<0.001**	−0.46 ± 0.92	1
**Posterior cingulate cortex**	−0.86 ± 0.4	1	−2.21 ± 0.84	0.768	−3.56 ± 0.74	**<0.001**	−2.45 ± 1.15	1
**Posterior orbitofrontal cortex**	−1.09 ± 0.31	**0.040**	−1.59 ± 0.64	1	−3.09 ± 0.57	**<0.001**	−0.93 ± 0.89	1
**Precentral gyrus**	−0.83 ± 0.34	1	−3.28 ± 0.71	**<0.001**	−3.43 ± 0.63	**<0.001**	‐0.8 ± 0.98	1
**Precuneus cortex**	−0.94 ± 0.35	0.664	−1.97 ± 0.73	0.649	−3.3 ± 0.65	**<0.001**	−1.52 ± 1.0	1
**Putamen**	−0.13 ± 0.27	1	−2.82 ± 0.57	**<0.001**	−2.64 ± 0.5	**<0.001**	−1.31 ± 0.78	1
**Rolandic operculum**	−0.39 ± 0.34	1	−2.01 ± 0.7	0.395	−3.5 ± 0.63	**<0.001**	−0.63 ± 0.97	1
**Superior occipital gyrus**	−1.33 ± 0.43	0.163	−1.81 ± 0.89	1	−3.53 ± 0.79	**<0.001**	−1.95 ± 1.22	1
**Superior parietal gyrus**	−1.54 ± 0.37	**0.003**	−1.1 ± 0.78	1	−1.85 ± 0.69	0.661	0.26 ± 1.06	1
**Superior temporal gyrus**	−0.67 ± 0.32	1	−1.83 ± 0.67	0.574	−3.16 ± 0.59	**<0.001**	−0.66 ± 0.92	1
**Superior temporal pole**	−0.71 ± 0.31	1	−0.01 ± 0.65	1	−2.75 ± 0.58	**<0.001**	−1.25 ± 0.89	1
**Supplementary motor area**	−0.6 ± 0.3	1	−2.86 ± 0.62	**<0.001**	−3.21 ± 0.55	**<0.001**	−0.99 ± 0.85	1
**Supramarginal gyrus**	−0.58 ± 0.33	1	−1.98 ± 0.69	0.373	−3.19 ± 0.61	**<0.001**	−1.37 ± 0.95	1
**Thalamus**	−0.67 ± 0.36	1	−3.19 ± 0.75	**0.002**	−2.69 ± 0.66	**0.005**	−3.58 ± 1.03	**0.050**

*Note*: *β* refers to the main effect of time from baseline. The interaction coefficients (*β_int_*) represent the difference in perfusion change over time between a given presymptomatic carrier group relative to non‐carrier controls. Coefficients reported as value ± standard error. *P* values for the main effect of time from baseline (*P*) and for the interaction effects (*P_int_*) were adjusted using Bonferroni correction, presented here. Bold emphasis has been placed on *P* values that are ≤ 0.05 after Bonferroni correction.

Abbreviations: *C9orf72*, chromosome 9 open reading frame 72; FTD, frontotemporal dementia; *GRN*, progranulin; *MAPT*, microtubule‐associated protein tau.

**TABLE 4:** Longitudinal region of interest analysis comparing cerebral perfusion between presymptomatic carriers of each major FTD mutation and non‐carrier controls modeled based on time from baseline and its interaction with carrier group within the right hemisphere.

Region			*C9orf72*		*GRN*		*MAPT*	
	*β*	*P*	*β_int_*	*P_int_*	*β_int_*	*P_int_*	*β_int_*	*P_int_*
**Amygdala**	−0.62 ± 0.27	1	−1.35 ± 0.57	1	−2.05 ± 0.5	**0.005**	−0.44 ± 0.78	1
**Angular gyrus**	−0.95 ± 0.39	1	−0.99 ± 0.82	1	−2.57 ± 0.72	**0.037**	−1.54 ± 1.12	1
**Anterior cingulate cortex**	−0.69 ± 0.34	1	−1.85 ± 0.7	0.801	−3.24 ± 0.63	**<0.001**	−0.18 ± 0.97	1
**Anterior orbitofrontal cortex**	−1.66 ± 0.35	**<0.001**	−1.88 ± 0.73	0.904	−2.56 ± 0.65	**0.007**	−0.15 ± 1.0	1
**Calcarine fissure**	−0.88 ± 0.42	1	−1.43 ± 0.88	1	−4.29 ± 0.78	**<0.001**	−2.34 ± 1.21	1
**Caudate nucleus**	−0.78 ± 0.27	0.330	−2.62 ± 0.56	**<0.001**	−2.03 ± 0.49	**0.004**	−0.94 ± 0.76	1
**Dorsolateral superior frontal gyrus**	−1.38 ± 0.33	**0.003**	−2.98 ± 0.69	**0.002**	−2.76 ± 0.61	**<0.001**	−1.18 ± 0.95	1
**Fusiform gyrus**	−0.55 ± 0.28	1	−0.75 ± 0.59	1	−2.7 ± 0.53	**<0.001**	−0.83 ± 0.82	1
**Gyrus rectus**	−0.44 ± 0.32	1	−0.98 ± 0.68	1	−3.24 ± 0.61	**<0.001**	0.17 ± 0.94	1
**Heschl's gyrus**	−0.89 ± 0.39	1	−2.35 ± 0.82	0.384	−2.93 ± 0.73	**0.006**	−1.01 ± 1.13	1
**Hippocampus**	−0.38 ± 0.28	1	−2.16 ± 0.6	**0.028**	−2.92 ± 0.53	**<0.001**	−1.23 ± 0.82	1
**Inferior frontal gyrus pars opercularis**	−0.7 ± 0.35	1	−3.49 ± 0.74	**<0.001**	−3.0 ± 0.66	**<0.001**	−2.41 ± 1.01	1
**Inferior frontal gyrus pars orbitalis**	−1.23 ± 0.37	0.075	−3.09 ± 0.77	**0.006**	−2.76 ± 0.68	**0.005**	−1.03 ± 1.05	1
**Inferior frontal gyrus pars triangularis**	−0.6 ± 0.34	1	−3.57 ± 0.72	**<0.001**	−3.07 ± 0.64	**<0.001**	−1.57 ± 0.98	1
**Inferior occipital gyrus**	−0.86 ± 0.44	1	−0.03 ± 0.93	1	−2.94 ± 0.82	**0.032**	−0.29 ± 1.27	1
**Inferior parietal gyrus**	−1.04 ± 0.39	0.710	−1.51 ± 0.82	1	−2.44 ± 0.73	0.074	−1.77 ± 1.12	1
**Inferior temporal gyrus**	−0.7 ± 0.28	1	−0.87 ± 0.59	1	−2.28 ± 0.52	**0.001**	−0.6 ± 0.81	1
**Insular cortex**	−0.61 ± 0.34	1	−3.58 ± 0.72	**<0.001**	−3.01 ± 0.64	**<0.001**	−1.89 ± 0.99	1
**Lateral orbitofrontal cortex**	−1.4 ± 0.42	0.088	−1.87 ± 0.84	1	−2.28 ± 0.81	0.476	0.02 ± 1.14	1
**Lingual gyrus**	−0.29 ± 0.35	1	−1.92 ± 0.74	0.870	−3.58 ± 0.65	**<0.001**	−1.77 ± 1.01	1
**Medial orbitofrontal cortex**	−1.09 ± 0.31	**0.036**	−2.17 ± 0.64	0.063	−2.94 ± 0.57	**<0.001**	0.19 ± 0.88	1
**Medial superior frontal gyrus**	−0.78 ± 0.31	1	−2.36 ± 0.65	**0.028**	−2.91 ± 0.58	**<0.001**	−0.64 ± 0.9	1
**Medial‐orbital superior frontal gyrus**	−1.05 ± 0.36	0.319	−1.45 ± 0.75	1	−3.52 ± 0.67	**<0.001**	0.52 ± 1.04	1
**Middle cingulate cortex**	−0.98 ± 0.32	0.204	−1.67 ± 0.67	1	−2.54 ± 0.6	**0.002**	−0.31 ± 0.92	1
**Middle frontal gyrus**	−1.12 ± 0.35	0.123	−3.59 ± 0.73	**<0.001**	−2.7 ± 0.65	**0.003**	−1.95 ± 1.0	1
**Middle occipital gyrus**	−1.33 ± 0.43	0.187	−0.52 ± 0.9	1	−2.11 ± 0.79	0.731	−2.01 ± 1.22	1
**Middle temporal gyrus**	−0.78 ± 0.32	1	−1.47 ± 0.68	1	−2.54 ± 0.6	**0.002**	−1.14 ± 0.93	1
**Middle temporal pole**	−0.37 ± 0.32	1	−0.39 ± 0.68	1	−2.18 ± 0.6	0.029	−1.05 ± 0.93	1
**Olfactory cortex**	−0.41 ± 0.33	1	−1.95 ± 0.7	0.466	−3.02 ± 0.62	**<0.001**	0.19 ± 0.97	1
**Paracentral lobule**	−0.78 ± 0.32	1	−2.29 ± 0.67	0.063	−2.53 ± 0.59	**0.002**	0.16 ± 0.91	1
**Parahippocampal gyrus**	−0.34 ± 0.28	1	−0.95 ± 0.58	1	−2.4 ± 0.52	**<0.001**	−0.75 ± 0.8	1
**Postcentral gyrus**	−1.08 ± 0.31	0.053	−1.86 ± 0.65	0.399	−1.77 ± 0.58	0.205	−1.1 ± 0.89	1
**Posterior cingulate cortex**	−1.05 ± 0.41	0.924	−2.76 ± 0.86	0.121	−2.92 ± 0.76	**0.012**	−2.35 ± 1.18	1
**Posterior orbitofrontal cortex**	−1.26 ± 0.32	**0.009**	−2.5 ± 0.68	**0.022**	−2.54 ± 0.6	**0.002**	−0.34 ± 0.93	1
**Precentral gyrus**	−0.9 ± 0.36	1	−3.1 ± 0.75	**0.003**	−2.55 ± 0.66	**0.011**	−1.75 ± 1.02	1
**Precuneus cortex**	−0.88 ± 0.35	1	−1.65 ± 0.74	1	−3.21 ± 0.65	**<0.001**	−1.49 ± 1.01	1
**Putamen**	‐0.42 ± 0.27	1	‐2.9 ± 0.57	**<0.001**	‐1.79 ± 0.5	**0.038**	‐0.99 ± 0.78	1
**Rolandic operculum**	−0.54 ± 0.33	1	−2.69 ± 0.7	**0.011**	−2.76 ± 0.62	**<0.001**	−1.68 ± 0.95	1
**Superior occipital gyrus**	−1.47 ± 0.43	0.064	−0.17 ± 0.91	1	−3.09 ± 0.8	**0.012**	−1.98 ± 1.24	1
**Superior parietal gyrus**	−1.25 ± 0.36	0.055	−1.57 ± 0.76	1	−1.81 ± 0.67	0.654	−0.36 ± 1.04	1
**Superior temporal gyrus**	−0.48 ± 0.33	1	−2.7 ± 0.69	**0.010**	−2.54 ± 0.61	**0.003**	−1.7 ± 0.95	1
**Superior temporal pole**	−0.8 ± 0.31	0.918	−1.76 ± 0.65	0.645	−2.35 ± 0.58	**0.005**	−0.78 ± 0.9	1
**Supplementary motor area**	−0.51 ± 0.3	1	−2.35 ± 0.63	**0.021**	−2.86 ± 0.56	**<0.001**	−0.59 ± 0.87	1
**Supramarginal gyrus**	−0.69 ± 0.36	1	−1.62 ± 0.76	1	−2.3 ± 0.67	0.061	−1.99 ± 1.04	1
**Thalamus**	−0.34 ± 0.35	1	−3.09 ± 0.73	**0.003**	−3.58 ± 0.65	**<0.001**	−2.89 ± 1.01	0.381

*Note*: β refers to the main effect of time from baseline. The interaction coefficients (*β_int_*) represent the difference in perfusion change over time between a given presymptomatic carrier group relative to non‐carrier controls. Coefficients reported as value ± standard error. *P* values for the main effect of time from baseline (*P*) and for the interaction effects (*P_int_*) were adjusted using Bonferroni correction, presented here. Bold emphasis has been placed on *P* values that are ≤ 0.05 after Bonferroni correction.

Abbreviations: *C9orf72*, chromosome 9 open reading frame 72; FTD, frontotemporal dementia; *GRN*, progranulin; *MAPT*, microtubule‐associated protein tau.

The main effect of time from baseline was significant in certain ROIs and demonstrated a trend of hypoperfusion occurring in participants as a function of time. However, the interaction effects between time from baseline and presymptomatic genetic subgroups were generally far more pronounced in one or more of the presymptomatic genetic subgroups than this main effect alone. Additionally, the coefficients for the main effects of presymptomatic genetic subgroups within the interaction model were not significant (*P* > 0.05) in any region and therefore not presented in Table 3 or Table 4.

Presymptomatic *C9orf72* carriers featured prominent changes in longitudinal perfusion in the frontal lobe; certain subcortical structures such as the caudate, putamen, and hippocampus; as well as the thalamus (Tables 3 and 4). Interestingly, while most significant regions were bilateral, a slight rightward asymmetry was observable across the inferior frontal lobe, with significant regions coming from the pars opercularis, pars orbitalis, pars triangularis, and orbitofrontal cortical regions, as well as the right superior temporal gyrus.

Coinciding with the global gray matter changes in perfusion, the presymptomatic *GRN* carriers featured more prominent decreases in perfusion over time relative to controls in a far more widespread manner, with nearly all regions demonstrating a significant effect. Notably, a leftward asymmetric effect is evident, as left hemispheric regions tended to demonstrate stronger effects based on *t* values and interaction coefficients than in similar right hemispheric regions (Tables 3 and 4).

*MAPT* presymptomatic carriers only showed significant decreases in perfusion over time in the left thalamus, an area that was also significant for *C9orf72* as well as *GRN* carriers, highlighting a region that is commonly affected in all genetic subgroups of FTD. No regions featured any significant increase in perfusion in any presymptomatic genetic subgroup relative to non‐carrier controls.

### 3.5 Perfusion in presymptomatic carriers beyond their expected year of symptom onset versus converters

Mutation‐positive converters pooled across genetic subgroups (*n* = 19; *C9orf72* = 7, *GRN *= 8, *MAPT* = 4) demonstrated a significant decline in perfusion from baseline based on their last follow‐up scan compared to presymptomatic carriers who went beyond their expected age of symptom onset (*n* = 22; *C9orf72* = 6, *GRN *= 14, *MAPT* = 2) without showing symptoms or signs of FTD (Table 5). These regions included the right middle frontal gyrus, inferior frontal gyrus pars triangularis, dorsolateral superior frontal gyrus, and posterior orbitofrontal cortex.

**TABLE 5:** Analysis of covariance results for converters versus presymptomatics past their expected year of disease onset.

Region of interest	Between‐groups delta	*F* value	*P* value uncorrected	Partial eta squared
**Right middle frontal gyrus**	−11.73	12.02	0.001	0.25
**Right inferior frontal gyrus pars triangularis**	−9.73	4.99	0.032	0.12
**Right dorsolateral superior frontal gyrus**	−8.28	4.78	0.035	0.11
**Right posterior orbitofrontal cortex**	−7.43	4.51	0.040	0.11
**Right thalamus**	−9.95	3.88	0.056	0.09
**Left postcentral gyrus**	−6.82	3.78	0.060	0.09
**Left paracentral lobule**	−9.14	3.71	0.063	0.11
**Left precentral gyrus**	−7.19	3.62	0.065	0.09
**Right medial superior frontal gyrus**	−6.63	2.98	0.093	0.07
**Left middle frontal gyrus**	−5.88	2.47	0.124	0.06
**Right insular cortex**	−7.00	2.46	0.126	0.06
**Right inferior frontal gyrus pars orbitalis**	−7.37	2.28	0.140	0.06
**Right inferior frontal gyrus Pars opercularis**	−6.95	2.04	0.162	0.05
**Right caudate nucleus**	−5.50	1.80	0.188	0.05
**Right precentral gyrus**	−6.18	1.58	0.217	0.04
**Left dorsolateral superior frontal gyrus**	−3.96	1.15	0.291	0.03
**Right hippocampus**	−4.87	1.07	0.307	0.03
**Right supplementary motor area**	−4.82	0.98	0.328	0.03
**Left thalamus**	−4.81	0.75	0.392	0.02
**Left medial superior frontal gyrus**	−3.03	0.67	0.417	0.02
**Right rolandic operculum**	−4.24	0.63	0.433	0.02
**Left insular cortex**	−3.20	0.60	0.443	0.02
**Right putamen**	−2.92	0.33	0.568	0.01
**Right superior temporal gyrus**	−2.75	0.28	0.600	0.01
**Left hippocampus**	1.35	0.23	0.633	0.01
**Left anterior cingulate cortex**	−1.55	0.14	0.710	0.00
**Left putamen**	0.85	0.09	0.767	0.00
**Left supplementary motor area**	−1.73	0.02	0.877	0.00
**Left caudate nucleus**	−0.67	0.01	0.924	0.00

*Note*: *F* values correspond to the main effect between the two groups. Between‐groups delta refers to the difference between the degree of perfusion decline in the converters vs. presymptomatics past their expected year of onset. Statistics for the covariates of age and sex have been omitted. Partial eta squared has been included as a measure of effect size.

## 4 DISCUSSION

This longitudinal study has identified specific patterns of perfusion decline in the most prevalent genetic subsets of FTD (i.e., *C9orf72*, *GRN*, and *MAPT*) at the presymptomatic stage. We have found that all genetic subgroups feature a more significant degree of global gray matter perfusion decrease over time relative to healthy controls. This contrasted with a structural analysis examining global brain volumes, which only detected gray matter atrophy in the *C9orf72* group at baseline, in agreement with the prior literature,^15^, ^19^ and which did not detect significant differences in rates of global gray matter volume change over time between any of the studied genetic subgroups. This observation highlights additional gains provided by cerebral perfusion measures longitudinally in the study of presymptomatic genetic FTD on top of volumetric analysis alone. Post hoc analysis further identified that significant hypoperfusion is detectable as early as year 1 follow‐up in *GRN* and *MAPT* subsets, and in *C9orf72* by year 2 follow‐up. Given that metrics typically used within FTD clinical practice could not differentiate presymptomatic groups from controls, these findings are strongly attributable to the effect of the disease mutation over nuisance factors such as age alone. Another key result was that the presymptomatic genetic subgroups featured their own regional patterns of perfusion decline, with *C9orf72* being focused around the frontal lobe with a slight right hemispheric bias, *GRN* featuring a more global and left hemispheric bias, and *MAPT* restricted to the left thalamus. These observations suggest the utility of longitudinal cerebral perfusion as an imaging biomarker differentiating between the major genetic subgroups of FTD at this presymptomatic disease stage. We also identified that conversion into the symptomatic FTD stage was associated with hypoperfusion in several right hemispheric frontal lobe regions. Our study is therefore the first to indicate the potential protective effects of maintaining regional cerebral perfusion and suggest its possible utility as a measure of drug efficacy in terms of slowing down FTD disease progression. Altogether, the findings of this study indicate that cerebral perfusion, as measured by ASL, has the characteristics of a promising biomarker for assessing disease progression in genetic FTD prior to symptom onset.

This body of work adds to the growing evidence that the salience network, which is involved in guiding behavior and attention, is fundamentally tied to FTD disease progression.^40^ Presymptomatic *C9orf72* and *GRN* groups demonstrated declining perfusion in several key component areas of this network, including the insula and anterior cingulate cortex, as well as the posterior orbitofrontal cortex, which projects into the network.^41^, ^42^ Both *C9orf72* and *GRN* mutations most commonly present with the behavioral variant of FTD (bvFTD), which is functionally related to the salience network. Within the GENFI cohort, most symptomatic individuals had a bvFTD presentation.^20^ Our perfusion findings are also consistent with observations of other genetic FTD neuroimaging studies that have identified connectivity reductions and gray matter atrophy in this network for presymptomatic and symptomatic individuals, respectively.^18^, ^19^, ^21^ Furthermore, it has been posited that von Economo neurons (VENs), cells that are concentrated within layer Vb of the of the cortex involved in salience network function, are particularly susceptible to pathology in the early stages of the FTD.^43^ Indeed, TAR DNA‐binding protein 43 (TDP‐43) proteinopathy, which is the dominant inclusion in *C9orf72* and *GRN* subsets of genetic FTD,^5^ has been detected within right frontoinsular VENs and is associated with salience network atrophy.^44^ Within this study, the right hemispheric posterior orbitofrontal regions also showed significant hypoperfusion in converters compared to non‐converters who went beyond their EYO, the bulk of which were *C9orf72* and *GRN* individuals. In an earlier GENFI analysis of mutation carriers versus non‐carriers, the insula featured neuroanatomical differences as early as 25 and 15 years prior to symptom onset for *C9orf72* and *GRN* carriers, respectively.^20^ From this collective evidence, we speculate that in *C9orf72* and *GRN* genetic subgroups, there is a TDP‐43–based neurodegenerative mechanism at play which targets the salience network at very early stages of the disease, with initial proteinopathy burden manifesting as local functional changes (hypoperfusion and connectivity loss) before translating into gross structural atrophy, and finally presenting as bvFTD in the clinic. We also speculate that certain genetic subgroups may exhibit either leftward or rightward frontal bias for the salience network, as the latter was evident in the *C9orf72* subset of this study and is consistent with prior imaging and pathological case series.^45^, ^46^ Future longitudinal neuroimaging studies will need to incorporate a complex multimodal approach on the same cohort to assess these conjectures.

The observation that the rate of global gray matter perfusion decline was most prominent in *GRN* and that it featured hypoperfusion relative to controls as early as 1 year after baseline measurement coincides with observed atrophy rates of this FTD genotype relative to others.^47^ Indeed, there is some evidence for disease acceleration to be more prominent in non‐tau variants, which would coincide with both *GRN* and *C9orf72* genotype groups featuring steeper global gray matter perfusion declines compared to *MAPT*.^48^ This widespread hypoperfusion seen in presymptomatic *GRN* carriers has been previously noted by Dopper et al.^12^ However, it is also important to note that this *GRN* group has the most statistical power within GENFI and had the largest number of follow‐up visits, which increases the probability of Type 1 statistical errors, even considering stringent multiple‐testing correction, such as Bonferroni's method. As in the Dopper et al. study,^12^ our results also demonstrate a left‐hemisphere asymmetry in terms of hypoperfusion effect size, which is in line with previous literature observing an asymmetric impact on the brain for this genotype.^17^, ^20^, ^49^ This may link to prior findings that *GRN* carriers also present as non–fluent‐variant primary progressive aphasia (nfvPPA),^50^ which is associated with atrophy and metabolic/perfusion decline involving the left frontal region.^51^ One of the most prominent regions of hypometabolism within the *GRN* subgroup was in the left inferior frontal lobe pars opercularis, a region considered to have the earliest involvement in nfvPPA.^50^ This region constitutes the main portion of Broca's speech area, which is primarily associated with the motor aspects of language production.^52^ Our hypoperfusion results in the *GRN* subgroup are also consistent with nfvPPA presenting with executive dysfunction alongside hypometabolism seen in the orbitofrontal cortex, anterior cingulate cortex, insula, precentral and postcentral gyrus, and thalamus.^53^

The single common area of hypoperfusion observed across all presymptomatic genetic FTD subgroups was the thalamus. The thalamus is a complex association of 50 to 60 subnuclei that serves as a central signal‐integration hub interconnected with networks that pass motor, visual, auditory, and somatosensory information to various cortical destinations.^54^ While initial reports demonstrated atrophy of this structure in *C9orf72* carriers,^19^ there have since been updates in the literature showing that both *GRN* and *MAPT* also feature thalamic atrophy.^55^, ^56^ *Post mortem* analyses confirm the thalamus is impacted in FTD,^56^, ^57^, ^58^ with one study finding that, compared to controls, patients with tau pathology showed similar degrees of thalamic volume reductions to those with TDP‐43 pathology.^55^ Within the GENFI cohort itself, a neuroanatomical study found that thalamic volume reduction was evident in *C9orf72* subjects at the presymptomatic stage, and in both *GRN* and *MAPT* subjects by the time they scored ≥1 on CDR plus NACC FTLD.^59^ These findings support the proposition that FTD may progress along large‐scale white matter networks,^60^ occurring at different rates for each genotype. Under such a hypothesis, it would not be surprising that one of the most interconnected regions of the brain features some perfusion decline in all FTD genetic subgroups. There is some evidence to suggest that subregions of the thalamus are relevant neuroanatomical structures in delineating variants of FTD, as seen in a meta‐analysis of studies reporting on volume reductions in thalamic subregions, with differing patterns across phenotypes, genotypes, and identified pathology.^56^ However, given the currently limited spatial resolution of ASL, only the overall left and right hemisphere equivalents of the thalamus could be reliably investigated.

This multicenter study is the largest longitudinal cerebral perfusion analysis in genetic FTD to date and assesses perfusion changes over time within all major genetic subgroups of FTD. The ASL analysis pipeline used was selected due to its adherence to ASL processing standards and ability to adjust for multicenter scanner, sequence, and software sources of variability.^26^, ^29^ Site and acquisition effects were also corrected for with the biasfield intensity normalization semi‐automatic spatial coefficient of variation quality control.^13^, ^31^ Gray matter atrophy effects were accounted for through robust regression‐based partial volume correction.^34^ Sensitivity analyses confirmed that individuals who were excluded due to these corrective measures were not significantly different by demographic and clinical measures. The decline in perfusion was also detectable in non‐carrier controls in global gray matter, albeit to a lesser extent than in mutation carriers, and this is in agreement with ASL studies of aging in healthy populations.^39^, ^61^, ^62^ This study is not without its limitations. We were unable to account for several variables that are known to contribute both to inter‐ and intra‐individual perfusion variation over time, including diurnal effects, caffeine consumption, post‐prandial status, among other factors.^63^ Additionally, the fewer number of follow‐up visits and participants in the *MAPT* subgroup may have contributed to the less steep rate of perfusion decline observed in whole brain gray matter analysis for that subset versus *C9orf72* or *GRN*. Finally, not all scanners were able to accommodate measuring perfusion within the cerebellum, a region that has been noted to feature some atrophy in *C9orf72* carriers.^15^, ^19^

To conclude, this study has demonstrated that cerebral perfusion carries the characteristics of a potential biomarker for FTD. It differentiated all presymptomatic carriers from non‐carriers, delineated variants of the disease from one another in terms of the regional pattern of perfusion decline, and showed promise in highlighting regions that feature the greatest change for participants who converted into a FTD phenotype. Ultimately, we hope that these results will not only further elucidate mechanisms leading to FTD that take place at the presymptomatic stage, but also facilitate effective therapeutic trial design to slow or even prevent FTD‐related neurodegeneration.^7^

## CONFLICT OF INTEREST STATEMENT

M.M. holds additional grants unrelated to this work from the Ontario Brain Institute, Washington University, as well as the Women's Brain Health Initiative and Brain Canada as part of the EU Joint Program for Neurodegenerative Disease Research; has clinical trials contracts with Roche and Alector; has received consulting fees from Ionis, Alector, Biogen Canada, Wave Life Science, Eisai Canada, and Novo Nordisk Canada; received royalties from the Henry Stewart Talks; has received payments from MINT Memory Clinics and ECHO Dementia Series; and holds unpaid Scientific Advisory Board roles with Alzheimer's Society Canada and Parkinson Canada. A.P.S. is a member on the Board of Directors for Parkinson Canada and the Canadian Academy of Health Sciences. B.B. is waiting on a patent on therapeutic intervention in genetic frontotemporal dementia and has received personal fees from UCB, Lilly, AviadoBio, and Denali. C.G. has received payment from Demensdagarna Örebro 2023, Diakonia Ersta sjukhus, and Göteborgsregionen 2023 and is involved as a leader of the Swedish FTD Initiative. D.C. received support from Alzheimer's Research UK (ARUK‐PG2017‐1946), the UCL/UCLH NIHR Biomedical Research Centre, and the UK Dementia Research Institute, which receives its funding from DRI Ltd; and holds a chair position in the Alzheimer's Association Neuroimaging Professional Interest Area. D.T.W. holds an unpaid medical advisory board member position for Hydrocephalus Canada. F.M. holds grants from the Tau Consortium (#A1133749), and the Carlos III Health Institute (PI19/01637). I.S. has participated on the board of Novo Nordisk. J.L. has received support from the DFG German Research Foundation under Germany's Excellence Strategy within the framework of the Munich Cluster for Systems Neurology (EXC 2145 SyNergy – ID 390857198); has received personal fees from EISAI and Biogen; has received payments from Abbview, Bayer Vital, Biogen, EISAI, TEVA, Roche, and Zambon; and is on an advisory board for Axon Neuroscience. J.P. holds grants with the Eurostars‐2 joint programme with co‐funding from the European Union Horizon 2020 research and innovation program (ASPIRE E!113701), the Dutch Heart Foundation (2020T049), the EU Joint Program for Neurodegenerative Disease Research, provided by the Netherlands Organisation for Health Research and Development and Alzheimer Nederland DEBBIE (JPND2020‐568‐106), and the Czech Health Research Council (NU23‐08‐00460). J.B.R. has received consulting fees from Astex, Curasen, UCB, WAVE, Prevail, and SVHealth; and is a participant on the board of Asceneuronl, an Associate Director at the Dementias Platform UK, and a Medical Advisor to both Cumulus Neuro and Astronautx. L.L.R. received support from the Guarantors of Brain and Alzheimer's Research UK. M.S. has received payments from UCB, Prevail, Ionis, Orphazyme, Servier, Reata, AviadoBio, GenOrph, Biohaven, Zavra, and Lilly. M.C.T. has received support from NIH and the Weston Brain Foundation; holds positions as a scientific advisor in the Women's Brain Project, Brain Injury Canada, and PSP Canada; and is involved in clinical trials conducted by Janssen, Biogen, Avanex, Green Valley, and Roche. M.F. is listed on a patent related to methods and kits for differential diagnosis of Alzheimer's disease versus frontotemporal dementia using blood biomarkers. J.D.R. participated on advisory boards for Aviado Bio, Arkuda Therapeutics, Prevail Therapeutics, Denali, and Wave Life Sciences. R.S.V. has received additional support from Sage Pharmaceuticals, outside the present study; has received consulting fees from Ionis, AviadoBio, Novo Nordisk, Pfizer, and Lilly; has received payments from Neuraxpharma and Roche Diagnostics; has received travel support from Esteve; and participates on the board of Wave Pharmaceuticals. R.V. is in contract with Alector, Denali, Eli Lilly, J&J, UCB, and Biogen; and participates on the boards of AC Immune, and Novartis. S.E.B. has received contracts from Genentech, Optina, Roche, Eli Lilly, Eisa/Biogen Idec, Novo Nordisk, Lilly Avid, and ICON; has received consulting fees from Roche, Biogen, Novo Nordisk, Eisai, and Eli Lilly; has received payments from Biogen, Roche New England Journal Manuscript, Roche Models of Care Analysis in Canada, and Eisai; and has participated on the boards of the Conference Board of Canada, World Dementia Council, University of Rochester Contribution to the Mission and Scientific Leadership of the Small Vessel VCID Biomarker Validation Consortium, and the National Institute of Neurological Disorders and Stroke. S.D. has been sponsored by Biogen, Novo Nordisk, Janssen, Alnylam, Wave Life Sciences, and Passage Bio; has received consulting fees from Eisai, QuRALIS, AI Therapeutics, and Eli Lilly; has received payments from Eisai; and has participated in the boards of IntelGenX and AVIADO Bio. M.O. reports receiving funding from BMBF – FTLD Consortium, the ALS Association, and EU‐MIRAIDE; has received consulting fees from Biogen, Axon, Roche, and Grifols; has patents with Foundation state Baden‐Wuerttemberg for beta‐Syn as a biomarker for neurodegenerative diseases; and participates on the Biogen ATLAS trail board; is a speaker for the FTLD consortium, is involved in an unpaid role with the German Society for CSF Diagnostics and Neurochemistry, and is involved without pay with the Society for CSF Diagnostics and Neurochemistry. M.P., S.M., N.L., H.J.J.M.M., D.T., M.B., S.B.M., E.R., A.B., J.C.vS., L.C.J., H.S., R.L., P.T., D.G., E.F., S.S., A.dM., C.B., A.G., and B.J.M. report no conflicts. Author disclosures are available in the supporting information.

## CONSENT STATEMENT

Ethical review boards from all sites approved the study protocol and all participating individuals provided written and informed consent in agreement with the Declaration of Helsinki.

## Supporting information

Supporting Information

Supporting Information
