# Supplementary material for: Longitudinal cerebral perfusion in presymptomatic genetic frontotemporal dementia: GENFI results
Source: Alzheimers Dement. 2024 Apr 16;20(5):3525–42. [Article in Italian] doi: 10.1002/alz.13750 (PMC11095434; doi:10.1002/alz.13750)
Supplement: Supplementary file 1 — Supporting Information [file ALZ-20-3525-s001.pdf]

Supplementary Material

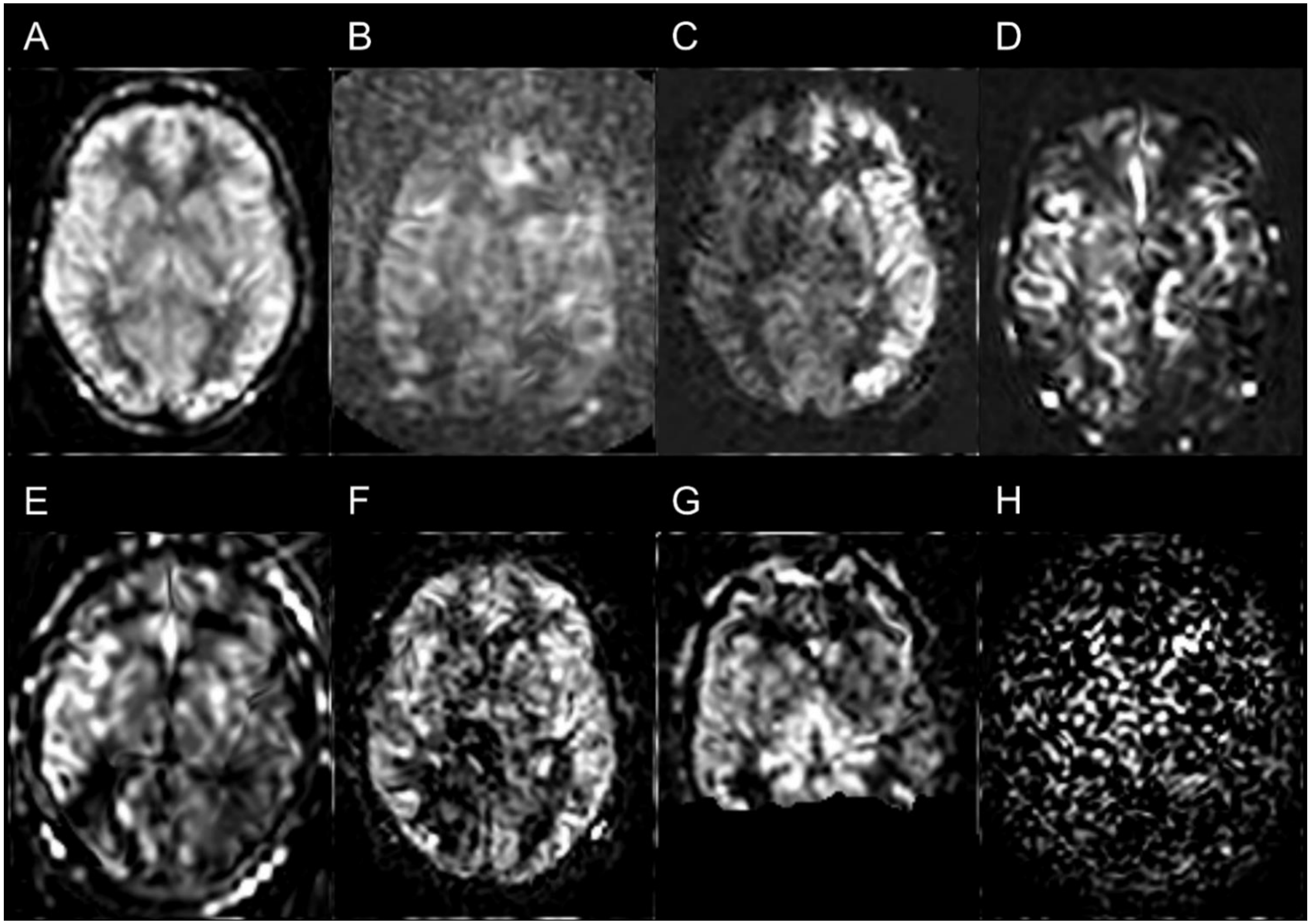

Figure S1 Axial views of processed cerebral perfusion volume examples during quality control. (A) An accepted image. Subsequent examples demonstrate excluded perfusion volumes based on (B) poor signal-to-noise, (C) uneven labeling, (D) excessive macrovascular signal, (E) poor coregistration, (F) artifacts, (G) clipping, (H) complete failure to process ASL timeseries into a perfusion volume by the ExploreASL pipeline.

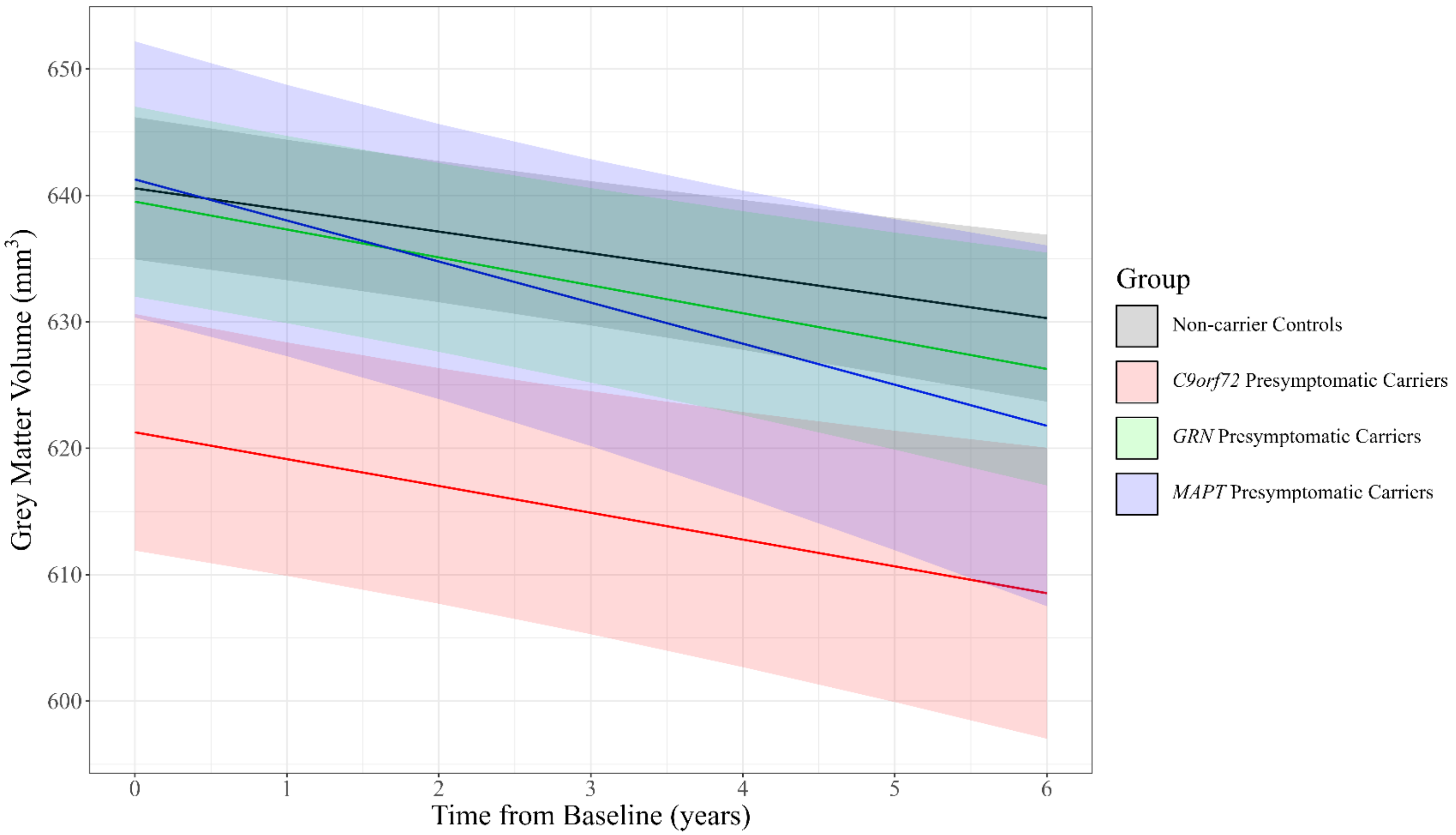

Figure S2 Mixed effects interaction plot of whole brain grey matter volume as a function of time from baseline assessment for non-carrier controls (black) versus presymptomatic carriers of mutations *C9orf72* (orange), *GRN* (green), and *MAPT* (cyan). Shaded areas represent 95% confidence intervals. The omnibus test for the interaction term resulted in a *P*-value of 0.5, indicating no significant differences between the slopes of group lines representing grey matter volume over time.

Table S1 - Summary of ASL acquisitions present within the dataset.

Manufacturer	Manufacturer Model	Pulse Sequence Type	ASL Context	Total Acquired Pairs	M0 Type	Average TR/TE (s/s)	ASL Type	Post Labeling Delay (s)	Labeling Duration (s)	Bolus Cut Off Technique	Bolus Cut Off Delay Times (s)	Background Suppression Timings (s)
GE	Discovery MR750	3D Spiral	deltam, m0scan	-	Included	4.632 / 0.010536	PCASL	1.525	1.45	-	-	1.465, 2.1, 2.6, 2.88
Philips	Achieva	2D EPI	cbf	-	Absent	4-4.3 / 0.011-0.012	PCASL	1.2	1.65	-	-	-
	Achieva	2D EPI	control-label alternating pairs	40	Absent	4 / 0.01-0.0128	PCASL	1.2	1.65	-	-	-
	Achieva	2D EPI	control-label alternating pairs	37	Absent	4.3 / 0.01-0.0128	PCASL	1.2	1.65	-	-	-
	Achieva dStream	2D EPI	control-label alternating pairs	40	Separate	4.02 / 0.0138-0.0156	PCASL	1.525	1.65	-	-	1.68, 2.83
Siemens	Skyra Fit	3D GRaSE	label-control alternating pairs	10	Absent	4 / 0.01322	PASL	1.99	-	QUIPSSII	0.7	0.25, 1.476
	Skyra Syngo	3D GraSE	label-control alternating pairs	3	Absent	5 / 0.01802	PASL	1.7	-	QUIPSSII	0.7	0.25, 1.476
	Prisma Fit	3D GraSE	label-control alternating pairs	10	Separate	4 / 0.01322	PASL	2	-	QUIPSSII	0.8	0.25, 1.476
	Prisma Fit	3D GraSE	label-control alternating pairs	5	Separate	4 / 0.01224	PASL	2	-	QUIPSSII	0.71	0.249, 1.475
	Trio Tim	3D GRaSE	control-label alternating pairs	10	Separate	4 / 0.0147	PASL	2	-	Q2TIPS	0.8, 1.99	0.1, 0.8

Table S2 – Additional medical information on the GENFI cohort.

Characteristic	Group				*P-value*
	**Non-Carrier Controls**, *N* = 158	***C9orf72*** **Presymptomatic Carriers**, *N* = 42	***GRN*** **Presymptomatic Carriers**, *N* = 70	***MAPT*** **Presymptomatic Carriers**, *N* = 31	
**Seizures**					0.2
Absent	156 (99%)	39 (93%)	70 (100%)	31 (100%)	
Recent/Active	1 (0.6%)	1 (2.4%)	0 (0%)	0 (0%)	
Remote/Inactive	1 (0.6%)	2 (4.8%)	0 (0%)	0 (0%)	
**Stroke**					>0.9
Absent	157 (99%)	42 (100%)	70 (100%)	31 (100%)	
Recent/Active	0 (0%)	0 (0%)	0 (0%)	0 (0%)	
Remote/Inactive	1 (0.6%)	0 (0%)	0 (0%)	0 (0%)	
**Traumatic Brain Injury**					0.5
Absent	140 (89%)	39 (93%)	62 (89%)	30 (97%)	
Recent/Active	0 (0%)	0 (0%)	0 (0%)	0 (0%)	
Remote/Inactive	18 (11%)	3 (7.1%)	8 (11%)	1 (3.2%)	
**Hypertension**					0.5
Absent	146 (92%)	37 (88%)	62 (89%)	28 (90%)	
Recent/Active	10 (6.3%)	5 (12%)	5 (7.1%)	3 (9.7%)	
Remote/Inactive	2 (1.3%)	0 (0%)	3 (4.3%)	0 (0%)	
**Hypercholesterolemia**					0.5
Absent	146 (92%)	36 (86%)	62 (89%)	29 (94%)	
Recent/Active	7 (4.4%)	4 (9.5%)	7 (10%)	2 (6.5%)	
Remote/Inactive	5 (3.2%)	2 (4.8%)	1 (1.4%)	0 (0%)	
**Diabetes Mellitus**					0.4
Absent	155 (98%)	41 (98%)	67 (96%)	30 (97%)	
Recent/Active	1 (0.6%)	1 (2.4%)	3 (4.3%)	1 (3.2%)	
Remote/Inactive	2 (1.3%)	0 (0%)	0 (0%)	0 (0%)	
**Smoking**					0.6
Absent	96 (73%)	31 (84%)	41 (67%)	24 (80%)	

Recent/Active	17 (13%)	2 (5.4%)	11 (18%)	3 (10%)	
Remote/Inactive	18 (14%)	4 (11%)	9 (15%)	3 (10%)	
**Excess Alcohol Use**					0.7
Absent	126 (96%)	36 (97%)	60 (98%)	28 (93%)	
Recent/Active	3 (2.3%)	1 (2.7%)	1 (1.6%)	2 (6.7%)	
Remote/Inactive	2 (1.5%)	0 (0%)	0 (0%)	0 (0%)	
**Recreational Drug Use**					>0.9
Absent	122 (93%)	36 (97%)	57 (93%)	28 (93%)	
Recent/Active	5 (3.8%)	1 (2.7%)	2 (3.3%)	1 (3.3%)	
Remote/Inactive	4 (3.1%)	0 (0%)	2 (3.3%)	1 (3.3%)	
**Autoimmune Disease**					0.7
Absent	126 (96%)	36 (97%)	58 (95%)	28 (93%)	
Recent/Active	3 (2.3%)	1 (2.7%)	3 (4.9%)	1 (3.3%)	
Remote/Inactive	2 (1.5%)	0 (0%)	0 (0%)	1 (3.3%)	

Table S3 - Number of non-carrier controls and presymptomatic carriers stratified across mutation at baseline and each yearly follow-up.

	Timepoint						
	**Baseline**	**Followup 1**	**Followup 2**	**Followup 3**	**Followup 4**	**Followup 5**	***P*-value**
**Group**							0.69
Non-carrier Controls	158	158	67	28	13	2	
C9orf72 Presymptomatic Carriers	42	42	16	9	5	0	
GRN Presymptomatic Carriers	70	70	32	14	3	0	
MAPT Presymptomatic Carriers	31	31	15	3	1	0	

Reported *P*-value pertains to Fisher's Exact test for proportion of counts.

Table S4 - Comparisons of linear mixed effects models during model building.

Model Description	Number of Parameters	AIC	BIC	Log Likelihood	*P*-value
Minimal	12	5823	5879	-2899	
Minimal + CBF at Baseline	13	5399	5460	-2686	0
Minimal + Random Slope per Subject	14	5813	5879	-2892	0.003
Minimal + Subject Nested within Family Membership	13	5825	5886	-2899	1

The minimal linear mixed effects model involves the main effects of carrier group and time from baseline, as well as their interaction, along with fixed effects covariates of age at baseline and sex, as well as a random intercept clustered by the individual subjects. Additional models included adding perfusion at baseline as a fixed effect, adding a random slope per subject, or representing subjects as being nested within families based on recorded family membership. Akaike's Information Criterion, Bayes Information Criterion, and the log likelihood ratio test were used to ascertain the direction in which the minimal model would be expanded. *P*-values represent the outcome of the log likelihood ratio tests.

Table S5 - Number of presymptomatic carriers past their expected year of disease onset versus converters at baseline and each yearly follow-up.

	Timepoint				
	**Baseline**	**Followup 1**	**Followup 2**	**Followup 3**	***P*-value**
**Group**					0.12
Presymptomatic Carriers Past EYO	22	22	5	1	
Converters	11	11	8	3	

Reported *P*-value pertains to Fisher's Exact test for proportion of counts.

Table S6 - Demographic and clinical characteristics of excluded subjects.

Characteristic	**Retained Subjects**, N = 301	**Excluded Subjects**, N = 41	***P*-value**
**Age (years)**	45.3 ± 12.3	46.7 ± 11.8	0.48
**Education (years)**	14.9 ± 3.4	13.9 ± 3.4	0.10
**Sex**			0.004
Female	191 (63%)	16 (39%)	
Male	110 (37%)	25 (61%)	
**Handedness**			1
Right	274 (91%)	38 (93%)	
Left	24 (8%)	3 (7%)	
Other	3 (1%)	0 (0%)	
**CDR plus NACC FTLD Score (categories *)**	0 (IQR 0 – 0)	0 (IQR 0 – 0)	-
**FTD Rating Scale (/100)**	95.7 ± 8.2	90.2 ± 20.9	0.13
**Cambridge Behavioral Inventory (/180)**	5.0 ± 7.2	7.6 ± 13.9	0.25
**Mini-Mental State Examination (/30)**	29.3 ± 1.0	29.5 ± 1.0	0.16

Data are represented as either *n* (%), mean ± standard deviation, or median (IQR = interquartile range). *P*-values stem from t-test for continuous variables and χ^2 or Fisher's Exact tests for categorical variables, depending on whether all cells were greater than 5 or not, respectively.
* Clinical Dementia Rating plus National Alzheimer's Coordinating Center Behaviour and Language Domains Rating Scale categories: 0 (normal); 0.5 (very mild); 1 (mild); 2 (moderate); 3 (severe).

GENFI Consortium Member Affiliations

Author	Affiliation
Annabel Nelson	Department of Neurodegenerative Disease, Dementia Research Centre, UCL Queen Square Institute of Neurology, London, UK
Martina Bocchetta	Department of Neurodegenerative Disease, Dementia Research Centre, UCL Queen Square Institute of Neurology, London, UK
David Cash	Department of Neurodegenerative Disease, Dementia Research Centre, UCL Queen Square Institute of Neurology, London, UK
David L Thomas	Neuroimaging Analysis Centre, Department of Brain Repair and Rehabilitation, UCL Institute of Neurology, Queen Square, London, UK
Emily Todd	Department of Neurodegenerative Disease, Dementia Research Centre, UCL Queen Square Institute of Neurology, London, UK
Hanya Benotmane	UK Dementia Research Institute at University College London, UCL Queen Square Institute of Neurology, London, UK
Jennifer Nicholas	Department of Medical Statistics, London School of Hygiene and Tropical Medicine, London, UK
Kiran Samra	Department of Neurodegenerative Disease, Dementia Research Centre, UCL Queen Square Institute of Neurology, London, UK
Rachelle Shafei	Department of Neurodegenerative Disease, Dementia Research Centre, UCL Queen Square Institute of Neurology, London, UK
Carolyn Timberlake	Department of Clinical Neurosciences, University of Cambridge, Cambridge, UK
Thomas Cope	Department of Clinical Neuroscience, University of Cambridge, Cambridge, UK
Timothy Rittman	Department of Clinical Neurosciences, University of Cambridge, Cambridge, UK
Alberto Benussi	Centre for Neurodegenerative Disorders, Department of Clinical and Experimental Sciences, University of Brescia, Brescia, Italy
Enrico Premi	Stroke Unit, ASST Brescia Hospital, Brescia, Italy
Roberto Gasparotti	Neuroradiology Unit, University of Brescia, Brescia, Italy
Silvana Archetti	Biotechnology Laboratory, Department of Diagnostics, ASST Brescia Hospital, Brescia, Italy
Stefano Gazzina	Neurology, ASST Brescia Hospital, Brescia, Italy
Valentina Cantoni	Centre for Neurodegenerative Disorders, Department of Clinical and Experimental Sciences, University of Brescia, Brescia, Italy
Andrea Arighi	Fondazione IRCCS Ca’ Granda Ospedale Maggiore Policlinico, Neurodegenerative Diseases Unit, Milan, Italy; University of Milan, Centro Dino Ferrari, Milan, Italy
Chiara Fenoglio	Fondazione IRCCS Ca’ Granda Ospedale Maggiore Policlinico, Neurodegenerative Diseases Unit, Milan, Italy; University of Milan, Centro Dino Ferrari, Milan, Italy
Elio Scarpini	Fondazione IRCCS Ca’ Granda Ospedale Maggiore Policlinico, Neurodegenerative Diseases Unit, Milan, Italy; University of Milan, Centro Dino Ferrari, Milan, Italy
Giorgio Fumagalli	Fondazione IRCCS Ca’ Granda Ospedale Maggiore Policlinico, Neurodegenerative Diseases Unit, Milan, Italy; University of Milan, Centro Dino Ferrari, Milan, Italy
Vittoria Borracci	Fondazione IRCCS Ca’ Granda Ospedale Maggiore Policlinico, Neurodegenerative Diseases Unit, Milan, Italy; University of Milan, Centro Dino Ferrari, Milan, Italy
Giacomina Rossi	Fondazione IRCCS Istituto Neurologico Carlo Besta, Milano, Italy
Giorgio Giaccone	Fondazione IRCCS Istituto Neurologico Carlo Besta, Milano, Italy
Giuseppe Di Fede	Fondazione IRCCS Istituto Neurologico Carlo Besta, Milano, Italy
Paola Caroppo	Fondazione IRCCS Istituto Neurologico Carlo Besta, Milano, Italy
Pietro Tiraboschi	Fondazione IRCCS Istituto Neurologico Carlo Besta, Milano, Italy
Sara Prioni	Fondazione IRCCS Istituto Neurologico Carlo Besta, Milano, Italy
Veronica Redaelli	Fondazione IRCCS Istituto Neurologico Carlo Besta, Milano, Italy
David Tang-Wai	The University Health Network, Krembil Research Institute, Toronto, Canada
Ekaterina Rogaeva	Tanz Centre for Research in Neurodegenerative Diseases, University of Toronto, Toronto, Canada

Miguel Castelo-Branco	Faculty of Medicine, University of Coimbra, Coimbra, Portugal
Morris Freedman	Baycrest Health Sciences, Rotman Research Institute, University of Toronto, Toronto, Canada
Ron Keren	The University Health Network, Toronto Rehabilitation Institute, Toronto, Canada
Sandra Black	Sunnybrook Health Sciences Centre, Sunnybrook Research Institute, University of Toronto, Toronto, Canada
Sara Mitchell	Sunnybrook Health Sciences Centre, Sunnybrook Research Institute, University of Toronto, Toronto, Canada
Christen Shoesmith	Department of Clinical Neurological Sciences, University of Western Ontario, London, Ontario, Canada
Robart Bartha	Department of Medical Biophysics, The University of Western Ontario, London, Ontario, Canada; Centre for Functional and Metabolic Mapping, Robarts Research Institute, The University of Western Ontario, London, Ontario, Canada
Rosa Rademakers	Center for Molecular Neurology, University of Antwerp
Jackie Poos	Department of Neurology, Erasmus Medical Center, Rotterdam, Netherlands
Janne M. Papma	Department of Neurology, Erasmus Medical Center, Rotterdam, Netherlands
Lucia Giannini	Department of Neurology, Erasmus Medical Center, Rotterdam, Netherlands
Rick van Minkelen	Department of Clinical Genetics, Erasmus Medical Center, Rotterdam, Netherlands
Yolande Pijnenburg	Amsterdam University Medical Centre, Amsterdam VUmc, Amsterdam, Netherlands
Benedetta Nacmias	Department of Neuroscience, Psychology, Drug Research and Child Health, University of Florence, Florence, Italy
Camilla Ferrari	Department of Neuroscience, Psychology, Drug Research and Child Health, University of Florence, Florence, Italy
Cristina Polito	Department of Biomedical, Experimental and Clinical Sciences "Mario Serio", Nuclear Medicine Unit, University of Florence, Florence, Italy
Gemma Lombardi	Department of Neuroscience, Psychology, Drug Research and Child Health, University of Florence, Florence, Italy
Valentina Bessi	Department of Neuroscience, Psychology, Drug Research and Child Health, University of Florence, Florence, Italy
Michele Veldsman	Nuffield Department of Clinical Neurosciences, Medical Sciences Division, University of Oxford, Oxford, UK
Christin Andersson	Department of Clinical Neuroscience, Karolinska Institutet, Stockholm, Sweden
Hakan Thonberg	Center for Alzheimer Research, Division of Neurogeriatrics, Karolinska Institutet, Stockholm, Sweden
Linn Öijerstedt	Center for Alzheimer Research, Division of Neurogeriatrics, Department of Neurobiology, Care Sciences and Society, Bioclinicum, Karolinska Institutet, Solna, Sweden; Unit for Hereditary Dementias, Theme Aging, Karolinska University Hospital, Solna, Sweden
Vesna Jelic	Division of Clinical Geriatrics, Karolinska Institutet, Stockholm, Sweden
Paul Thompson	Division of Neuroscience and Experimental Psychology, Wolfson Molecular Imaging Centre, University of Manchester, Manchester, UK
Tobias Langheinrich	Division of Neuroscience and Experimental Psychology, Wolfson Molecular Imaging Centre, University of Manchester, Manchester, UK; Manchester Centre for Clinical Neurosciences, Department of Neurology, Salford Royal NHS Foundation Trust, Manchester, UK
Albert Lladó	Alzheimer's disease and Other Cognitive Disorders Unit, Neurology Service, Hospital Clínic, Barcelona, Spain
Anna Antonell	Alzheimer's disease and Other Cognitive Disorders Unit, Neurology Service, Hospital Clínic, Barcelona, Spain
Jaume Olives	Alzheimer's disease and Other Cognitive Disorders Unit, Neurology Service, Hospital Clínic, Barcelona, Spain
Mircea Balasa	Alzheimer's disease and Other Cognitive Disorders Unit, Neurology Service, Hospital Clínic, Barcelona, Spain
Nuria Bargalló	Imaging Diagnostic Center, Hospital Clínic, Barcelona, Spain
Sergi Borrego-Ecija	Alzheimer's disease and Other Cognitive Disorders Unit, Neurology Service, Hospital Clínic, Barcelona, Spain
Ana Verdelho	Department of Neurosciences and Mental Health, Centro Hospitalar Lisboa Norte - Hospital de Santa Maria & Faculty of Medicine, University of Lisbon, Lisbon, Portugal
Carolina Maruta	Laboratory of Language Research, Centro de Estudos Egas Moniz, Faculty of Medicine, University of Lisbon, Lisbon, Portugal

Catarina B. Ferreira	Laboratory of Neurosciences, Faculty of Medicine, University of Lisbon, Lisbon, Portugal
Gabriel Miltenberger	Faculty of Medicine, University of Lisbon, Lisbon, Portugal
Frederico Simões do Couto	Faculdade de Medicina, Universidade Católica Portuguesa
Alazne Gabilondo	Cognitive Disorders Unit, Department of Neurology, Donostia University Hospital, San Sebastian, Gipuzkoa, Spain; Neuroscience Area, Biodonostia Health Research Insitute, San Sebastian, Gipuzkoa, Spain
Ana Gorostidi	Neuroscience Area, Biodonostia Health Research Insitute, San Sebastian, Gipuzkoa, Spain
Jorge Villanua	OSATEK, University of Donostia, San Sebastian, Gipuzkoa, Spain
Marta Cañada	CITA Alzheimer, San Sebastian, Gipuzkoa, Spain
Mikel Tainta	Neuroscience Area, Biodonostia Health Research Insitute, San Sebastian, Gipuzkoa, Spain
Miren Zulaica	Neuroscience Area, Biodonostia Health Research Insitute, San Sebastian, Gipuzkoa, Spain
Myriam Barandiaran	Cognitive Disorders Unit, Department of Neurology, Donostia University Hospital, San Sebastian, Gipuzkoa, Spain; Neuroscience Area, Biodonostia Health Research Insitute, San Sebastian, Gipuzkoa, Spain
Patricia Alves	Neuroscience Area, Biodonostia Health Research Insitute, San Sebastian, Gipuzkoa, Spain; Department of Educational Psychology and Psychobiology, Faculty of Education, International University of La Rioja, Logroño, Spain
Benjamin Bender	Department of Diagnostic and Interventional Neuroradiology, University of Tübingen, Tübingen, Germany
Carlo Wilke	Department of Neurodegenerative Diseases, Hertie-Institute for Clinical Brain Research and Center of Neurology, University of Tübingen, Tübingen, Germany; Center for Neurodegenerative Diseases (DZNE), Tübingen, Germany
Lisa Graf	Department of Neurodegenerative Diseases, Hertie-Institute for Clinical Brain Research and Center of Neurology, University of Tübingen, Tübingen, Germany
Annick Vogels	Department of Human Genetics, KU Leuven, Leuven, Belgium
Mathieu Vandenbulcke	Geriatric Psychiatry Service, University Hospitals Leuven, Belgium; Neuropsychiatry, Department of Neurosciences, KU Leuven, Leuven, Belgium
Philip Van Damme	Neurology Service, University Hospitals Leuven, Belgium; Laboratory for Neurobiology, VIB-KU Leuven Centre for Brain Research, Leuven, Belgium
Rose Bruffaerts	Department of Biomedical Sciences, University of Antwerp, Antwerp, Belgium; Biomedical Research Institute, Hasselt University, 3500 Hasselt, Belgium
Koen Poesen	Laboratory for Molecular Neurobiomarker Research, KU Leuven, Leuven, Belgium
Pedro Rosa-Neto	Translational Neuroimaging Laboratory, McGill Centre for Studies in Aging, McGill University, Montreal, Québec, Canada
Serge Gauthier	Alzheimer Disease Research Unit, McGill Centre for Studies in Aging, Department of Neurology & Neurosurgery, McGill University, Montreal, Québec, Canada
Agnès Camuzat	Sorbonne Université, Paris Brain Institute – Institut du Cerveau – ICM, Inserm U1127, CNRS UMR 7225, AP-HP - Hôpital Pitié-Salpêtrière, Paris, France
Alexis Brice	Sorbonne Université, Paris Brain Institute – Institut du Cerveau – ICM, Inserm U1127, CNRS UMR 7225, AP-HP - Hôpital Pitié-Salpêtrière, Paris, France; Reference Network for Rare Neurological Diseases (ERN-RND)
Anne Bertrand	Sorbonne Université, Paris Brain Institute – Institut du Cerveau – ICM, Inserm U1127, CNRS UMR 7225, AP-HP - Hôpital Pitié-Salpêtrière, Paris, France; Inria, Aramis project-team, F-75013, Paris, France; Centre pour l'Acquisition et le Traitement des Images, Institut du Cerveau et la Moelle, Paris, France
Aurélie Funkiewiez	Centre de référence des démences rares ou précoces, IM2A, Département de Neurologie, AP-HP - Hôpital Pitié-Salpêtrière, Paris, France; Sorbonne Université, Paris Brain Institute – Institut du Cerveau – ICM, Inserm U1127, CNRS UMR 7225, AP-HP - Hôpital Pitié-Salpêtrière, Paris, France
Daisy Rinaldi	Centre de référence des démences rares ou précoces, IM2A, Département de Neurologie, AP-HP - Hôpital Pitié-Salpêtrière, Paris, France; Sorbonne Université, Paris Brain Institute – Institut du Cerveau – ICM, Inserm U1127, CNRS UMR 7225, AP-HP - Hôpital Pitié-Salpêtrière, Paris, France; Département de Neurologie, AP-HP - Hôpital Pitié-Salpêtrière, Paris, France
Dario Saracino	Sorbonne Université, Paris Brain Institute – Institut du Cerveau – ICM, Inserm U1127, CNRS UMR 7225, AP-HP - Hôpital Pitié-Salpêtrière, Paris, France; Inria, Aramis project-team, F-75013, Paris, France; Centre de référence des démences rares ou précoces, IM2A, Département de Neurologie, AP-HP - Hôpital Pitié-Salpêtrière, Paris, France
Olivier Colliot	Sorbonne Université, Paris Brain Institute – Institut du Cerveau – ICM, Inserm U1127, CNRS UMR 7225, AP-HP - Hôpital Pitié-Salpêtrière, Paris, France; Inria, Aramis project-team, F-75013, Paris, France; Centre pour l'Acquisition et le Traitement des Images, Institut du Cerveau et la Moelle, Paris, France

Sabrina Sayah	Sorbonne Université, Paris Brain Institute – Institut du Cerveau – ICM, Inserm U1127, CNRS UMR 7225, AP-HP - Hôpital Pitié-Salpêtrière, Paris, France
Catharina Prix	Neurologische Klinik, Ludwig-Maximilians-Universität München, Munich, Germany
Elisabeth Wlasich	Neurologische Klinik, Ludwig-Maximilians-Universität München, Munich, Germany
Olivia Wagemann	Neurologische Klinik, Ludwig-Maximilians-Universität München, Munich, Germany
Sandra Loosli	Neurologische Klinik, Ludwig-Maximilians-Universität München, Munich, Germany
Sonja Schönecker	Neurologische Klinik, Ludwig-Maximilians-Universität München, Munich, Germany
Tobias Hoegen	Neurologische Klinik, Ludwig-Maximilians-Universität München, Munich, Germany
Jolina Lombardi	Department of Neurology, University of Ulm, Ulm
Sarah Anderl-Straub	Department of Neurology, University of Ulm, Ulm, Germany
Adeline Rollin	CHU, CNR-MAJ, Labex Distalz, LiCEND Lille, France
Gregory Kuchcinski	Univ Lille, France; Inserm 1172, Lille, France; CHU, CNR-MAJ, Labex Distalz, LiCEND Lille, France
Maxime Bertoux	Inserm 1172, Lille, France; CHU, CNR-MAJ, Labex Distalz, LiCEND Lille, France
Thibaud Lebouvier	Univ Lille, France; Inserm 1172, Lille, France; CHU, CNR-MAJ, Labex Distalz, LiCEND Lille, France
Vincent Deramecourt	Univ Lille, France; Inserm 1172, Lille, France; CHU, CNR-MAJ, Labex Distalz, LiCEND Lille, France
Beatriz Santiago	Neurology Department, Centro Hospitalar e Universitario de Coimbra, Coimbra, Portugal
Diana Duro	Faculty of Medicine, University of Coimbra, Coimbra, Portugal
Maria João Leitão	Centre of Neurosciences and Cell Biology, Universidade de Coimbra, Coimbra, Portugal
Maria Rosario Almeida	Faculty of Medicine, University of Coimbra, Coimbra, Portugal
Miguel Tábuas-Pereira	Neurology Department, Centro Hospitalar e Universitario de Coimbra, Coimbra, Portugal
Sónia Afonso	Instituto Ciencias Nucleares Aplicadas a Saude, Universidade de Coimbra, Coimbra, Portugal
